# Reduced levels of hydroxylated, polyunsaturated ultra long-chain fatty acids in the serum of colorectal cancer patients: implications for early screening and detection

**DOI:** 10.1186/1741-7015-8-13

**Published:** 2010-02-15

**Authors:** Shawn A Ritchie, Pearson WK Ahiahonu, Dushmanthi Jayasinghe, Doug Heath, Jun Liu, Yingshen Lu, Wei Jin, Amir Kavianpour, Yasuyo Yamazaki, Amin M Khan, Mohammad Hossain, Khine Khine Su-Myat, Paul L Wood, Kevin Krenitsky, Ichiro Takemasa, Masakazu Miyake, Mitsugu Sekimoto, Morito Monden, Hisahiro Matsubara, Fumio Nomura, Dayan B Goodenowe

**Affiliations:** 1 Phenomenome Discoveries Inc, Saskatoon, SK, Canada; 2 Bioserve Biotechnologies Inc, Laurel, MD, USA; 3 Department of Surgery, Graduate School of Medicine, Osaka University, Osaka, Japan; 4 Department of Frontier Surgery, Graduate School of Medicine, Chiba University, Chiba, Japan; 5 Department of Molecular Diagnosis, Graduate School of Medicine, Chiba University, Chiba, Japan

## Abstract

**Background:**

There are currently no accurate serum markers for detecting early risk of colorectal cancer (CRC). We therefore developed a non-targeted metabolomics technology to analyse the serum of pre-treatment CRC patients in order to discover putative metabolic markers associated with CRC. Using tandem-mass spectrometry (MS/MS) high throughput MS technology we evaluated the utility of selected markers and this technology for discriminating between CRC and healthy subjects.

**Methods:**

Biomarker discovery was performed using Fourier transform ion cyclotron resonance mass spectrometry (FTICR-MS). Comprehensive metabolic profiles of CRC patients and controls from three independent populations from different continents (USA and Japan; total *n *= 222) were obtained and the best inter-study biomarkers determined. The structural characterization of these and related markers was performed using liquid chromatography (LC) MS/MS and nuclear magnetic resonance technologies. Clinical utility evaluations were performed using a targeted high-throughput triple-quadrupole multiple reaction monitoring (TQ-MRM) method for three biomarkers in two further independent populations from the USA and Japan (total *n *= 220).

**Results:**

Comprehensive metabolomic analyses revealed significantly reduced levels of 28-36 carbon-containing hydroxylated polyunsaturated ultra long-chain fatty-acids in all three independent cohorts of CRC patient samples relative to controls. Structure elucidation studies on the C28 molecules revealed two families harbouring specifically two or three hydroxyl substitutions and varying degrees of unsaturation. The TQ-MRM method successfully validated the FTICR-MS results in two further independent studies. In total, biomarkers in five independent populations across two continental regions were evaluated (three populations by FTICR-MS and two by TQ-MRM). The resultant receiver-operator characteristic curve AUCs ranged from 0.85 to 0.98 (average = 0.91 ± 0.04).

**Conclusions:**

A novel comprehensive metabolomics technology was used to identify a systemic metabolic dysregulation comprising previously unknown hydroxylated polyunsaturated ultra-long chain fatty acid metabolites in CRC patients. These metabolites are easily measurable in serum and a decrease in their concentration appears to be highly sensitive and specific for the presence of CRC, regardless of ethnic or geographic background. The measurement of these metabolites may represent an additional tool for the early detection and screening of CRC.

## Background

Colorectal cancer (CRC) mortality remains one of the highest among all cancers, second to only lung cancer (Canadian Cancer Statistics, 2008). Despite the known benefits of early detection, screening programmes based on colonoscopy and fecal occult blood testing have been plagued with challenges such as public acceptance, cost, limited resources, accuracy and standardization. There is consensus in the field that the use of colonoscopy alone for CRC screening is not practical [1], and that a minimally-invasive serum-based test capable of accurately identifying subjects who are high risk for the development of CRC would result in a higher screening compliance than current approaches and better utilization of existing endoscopy resources [1-3]. Although there have been multiple reports of altered transcript levels [4-11], aberrantly methylated gene products [12-14] and proteomic patterns [15-18] associated with biological samples from CRC patients, few if any have advanced into clinically useful tests. This may be due to a number of reasons including technical hurdles in assay design, challenges obtaining reproducible results, costs and lengthy regulatory processes. Furthermore, most of the tests currently used or in development are based upon the detection of tumour-specific markers and have poor sensitivity for identifying subjects who are either very early stage, or are predisposed to risk but show no clinical presentation of disease.

Although causal genetic alterations for CRC have been well characterized, the number of cases due to adenomatous (APC) and hereditary nonpolyposs colorectal cancer are less than 5% of the total, with approximately 15% claimed to be attributable to inheritable family risk likely due to complex patterns of low penetrance mutations which have yet to be delineated [19]. The fact remains that approximately 80% of CRC cases are thought to arise sporadically, with diet and lifestyle as key risk factors [20,21]. In addition, an individual's microbiome is intricately linked to their gastrointestinal physiological status and may itself be involved as a risk factor [22]. Given that metabolism is heavily influenced by both diet and lifestyle and that the microbiome contributes its own metabolic processes, it is surprising that there has been little effort aimed identifying metabolic markers as risk indicators of CRC. This may, in part, have been due to the lack of platform technologies and informatics approaches capable of comprehensively characterizing metabolites in a similar way that DNA microarrays or surface-enhanced laser desorption ionization can characterize transcripts or proteins, respectively.

Recently, however, there have been rapid advances made in mass spectrometric-based systems which can identify large numbers of metabolic components within samples in a parallel manner [23-25]. Fourier transform ion cyclotron resonance mass spectrometry (FTICR-MS) is based upon the principle that charged particles exhibit cyclotron motion in a magnetic field, where the spin frequency is proportional the mass [26]. FTICR-MS is known for its high resolving power and capability of detecting ions with mass accuracy below 1 part per million (ppm). Liquid sample extracts can be directly infused using electrospray ionization (ESI) and atmospheric pressure chemical ionization (APCI) without chromatographic separation [23], where ions with differing mass to charge (m/z) ratios can be simultaneously resolved using a Fourier transformation. Using informatics approaches, spectral files from multiple samples can be accurately aligned and peak intensities across the samples compared [23]. High resolution also enables the prediction of elemental composition of all ions detected in a sample, providing a solid foundation for metabolite classification and identification, as well as the ability to construct *de novo *metabolic networks [23,27]. The combination of liquid extraction, flow injection, high resolution and informatics affords a unique opportunity to broadly characterize the biochemical composition of samples, with no *a priori *knowledge about the sample itself, to a degree which was not previously possible. This 'non-targeted' approach has the advantage of detecting novel compounds and is therefore ideally suited for biomarker-driven discovery applications. Using a MS-based discovery platform for metabolic biomarker identification also has the added advantage of straightforward translation into a quantitative method based upon triple-quadruple multiple-reaction-monitoring (TQ-MRM), similar to the clinical methods used to screen for inborn errors of metabolism [28].

Here we report on the application of this technology for characterizing the serum metabolomes of treatment-naive CRC patients and healthy asymptomatic subjects. A specific metabolic perturbation was discovered in the serum of CRC patients compared to controls in three independent and unrelated sets of samples (total *n *of 222). We further verify the perturbation using a tandem MS (MS/MS) approach in two additional independent case-control populations totalling 220 subjects. Implications of the findings for CRC screening are discussed.

## Methods

### Patient sample selection

Clinical samples used for the first discovery project were obtained from Genomics Collaborative, Inc (GCI, MA, USA), while samples for the second discovery project and one validation project were obtained from Seracare Lifesciences (MA, USA). These companies specialize in the collection and storage of serum and tissue samples specifically for research purposes. Samples were collected, processed and stored in a consistent manner by teams of physicians as part of a global initiative using standardized protocols and operating procedures. Collection protocols for GCI and Seracare Lifesciences were approved by the Western Institutional Review Board and all samples were properly consented. The inclusion criterion for patient sample selection from the GCI and Seracare biobanks for both the discovery and validation cohorts was that the serum be taken prior to any form of treatment, including surgery, chemo or radiation therapies. All samples were accompanied by detailed pathology reports which were independently verified by certified pathologists at GCI and Seracare. The GCI discovery sample set included serum samples from 40 pre-treatment CRC patients and matched 50 controls; the Seracare discovery set included samples from 26 pre-treatment CRC and matched 25 controls, and the validation Seracare set included 70 pretreatment CRC and matched 70 controls. The discovery samples provided by Osaka Medical University (Osaka, Japan) included 46 pre-surgery CRC patients matched 35 controls which were prospectively collected according to the standard collection protocol of the institution and were properly consented. Study protocols were performed according to the ethical guidelines set by the committee of the three ministries of the Japanese Government. The samples for the Chiba, Japan, validation population, which included 40 pre-surgery CRC patients and 40 matched controls, were also prospectively consented and collected under an ethics reviewed protocol approved by the Institutional Review Board of Graduate School of Medicine, Chiba University. A summary of the populations including disease staging is shown in Table 1. All samples were processed and analysed in a randomized manner and the results unblinded following analysis.

**Table 1:** Summary of case-control populations used in this study

	FTICR-MS discovery						MRM validation			
		
	Genomics Collaborative		Seracare 1		Osaka		Chiba		Seracare 2	
	**CRC**	**Control**	**CRC**	**Control**	**CRC**	**Control**	**CRC**	**Control**	**CRC**	**Control**

**Total**	40	50	26	25	46	35	40	40	70	70
**Male *N***	19	24	17	16	27	-	19	24	44	41
**Male age**	59 (30-78)	56 (30-78)	62 (46-80)	51 (35-70)	63 (28-90)	-	68 (45-91)	48 (36-69)	67 (39-87)	63 (32-82)
**Male BMI**	20.9 ± 3.8	25.0 ± 0.9	24.3 ± 5.7	25.6 ± 4.6	NA	-	NA	NA	28.0 ± 4.8	26. ± 4.2
**Female N**	21	26	9	9	19	-	21	16	26	29
**Female age**	54 (40-82)	55 (40-79)	78 (59-86)	55 (26-95)	65 (31-77)	-	70 (51-84)	49 (39-59)	73 (35-90)	56 (26-86)
**Female BMI**	19.9 ± 4.6	24.8 ± 2.2	23 ± 3.2	29 ± 8.0	NA	-	NA	NA	25.5 ± 4.4	24.0 ± 4.5

**Stage 0/I**	8	-	5	-	10	-	9	-	13	-
**Stage II**	16	-	8	-	14	-	18	-	21	-
**Stage III**	15	-	8	-	12	-	11	-	25	-
**Stage IV**	1	-	2	-	8	-	2	-	7	-
**Unknown**	0	-	3	-	2	-	0	-	4	-

BMI = body mass index; CRC = colorectal cancer; FTICT-MS = Fourier transform ion cyclotron resonance mass spectroscopy; MRM = multiple reaction monitoring.

### Sample extraction

Serum samples were stored at -80°C until thawed for analysis and were only thawed once. All extractions were performed on ice. Serum samples were prepared for FTICR-MS analysis by first sequentially extracting equal volumes of serum with 1% ammonium hydroxide and ethyl acetate (EtOAc) three times. Samples were centrifuged between extractions at 4°C for 10 min at 3500 rpm and the organic layer removed and transferred to a new tube (extract A). A 1:5 ratio of EtOAc (extract A) to butanol (BuOH) was then evaporated under nitrogen to the original BuOH starting volume (extract B). All extracts were stored at -80°C until FTICR-MS analysis.

### FTICR-MS analysis

For analysis under negative ESI conditions, sample extract B was diluted 10-fold in methanol:0.1% (v/v) ammonium hydroxide (50:50, v/v) prior to direct infusion. For APCI, extract A was directly injected without diluting. All analyses were performed on a Bruker Daltonics APEX III FTICR-MS equipped with a 7.0 T actively shielded superconducting magnet (Bruker Daltonics, MA, USA). Samples were directly injected using ESI and APCI at a flow rate of 600 μL per hour. Ion transfer/detection parameters were optimized using a standard mix of serine, tetra-alanine, reserpine, Hewlett-Packard tuning mix and the adrenocorticotrophic hormone fragment 4-10. In addition, the instrument conditions were tuned to optimize ion intensity and broad-band accumulation over the mass range of 100-1000 atomic mass units (amu) according to the instrument manufacturer's recommendations. A mixture of the above mentioned standards was used to internally calibrate each sample spectrum for mass accuracy over the acquisition range of 100-1000 amu. FTICR data were analysed using a linear least-squares regression line, mass axis values were calibrated such that each internal standard mass peak had a mass error of < 1 part ppm compared with its theoretical mass. Using XMASS software from Bruker Daltonics Inc (CA, USA), data file sizes of one megaword were acquired and zero-filled to two megawords. A SINm data transformation was performed prior to Fourier transform and magnitude calculations. The mass spectra from each analysis were integrated, creating a peak list that contained the accurate mass and absolute intensity of each peak. Compounds in the range of 100-1000 mz were analysed. In order to compare and summarize the data, all detected mass peaks were converted to their corresponding neutral masses, assuming hydrogen adduct formation. A self-generated two-dimensional (mass versus sample intensity) array was then created using *DISCOVA*metrics™ software (Phenomenome Discoveries Inc, Saskatoon, Canada). The data from multiple files were integrated and this combined file was then processed in order to determine all of the unique masses. The average of each unique mass was determined, representing the *y*-axis. A column was created for each file that was originally selected to be analysed, representing the *x*-axis. The intensity for each mass found in each of the files selected was then filled into its representative *x,y *coordinate. Coordinates that did not contain an intensity value were left blank. Each of the spectra was then peak-picked in order to obtain the mass and intensity of all metabolites detected. The data from all modes were then merged to create one data file per sample. The data from all 90 discovery serum samples were then merged and aligned to create a two-dimensional metabolite array in which each sample is represented by a column, each unique metabolite is represented by a single row and each cell in the array corresponds to a metabolite intensity for a given sample. The array tables were then used for statistical analysis described in 'statistical analyses' (see Additional File 1).

### Full-scan quadruple time-of-flight (Q-TOF) and high performance liquid chromatography (HPLC)-coupled MS/MS spectrometry

Ethyl acetate extracts from five CRC and five normal samples were evaporated under nitrogen gas and reconstituted in 70 μL of isopropanol:methanol:formic acid (10:90:0.1). Ten microlitres of the reconstituted sample was subjected to HPLC (HP 1100 with Hypersil ODS 5 μm, 125 × 4 mm column; Agilent Technologies, CA, USA) for full scan and 30 μL for MS/MS at a flow rate of 1 mL/min. Eluate from the HPLC was analysed using an ABI QSTAR^® ^XL mass spectrometer fitted with an APCI source in negative mode. The scan type in full scan mode was TOF with an accumulation time of 1.0000 s, mass range between 50 and 1500 Da and duration time of 55 min. Source parameters were as follows: ion source gas (GS) 1 80; ion GS2 10; curtain gas (CUR) 30; nebulizer current (NC) -3.0; temperature 400°C; declustering potential (DP) -60; focusing potential (FP) -265; DP2 -15. In MS/MS mode, scan type was product ion, accumulation time was 1.0000 s, scan range between 50 and 650 Da and duration time 55 min. All source parameters are the same as above, with collision energy (CE) of -35 V and collision gas (CID, nitrogen) of 5 psi. For MS3 work, the excitation energy was set at 180 V.

### Preliminary isolation of CRC biomarkers and NMR analysis

For the thin layer chromatographic methods, all chemicals and media were purchased from Sigma-Aldrich Canada Ltd (ON, Canada). All solvents were HPLC grade. Analytical thin layer chromatography (TLC) was carried out on pre-coated silica gel TLC aluminum sheets (EM Science, NJ, USA; Kieselgel 60 F_254_, 5 × 2 cm × 0.2 mm). Compounds were visualized under ultraviolet light (254/366 nm) or placed in an iodine vapour tank and by dipping the plates in a 5% aqueous (w/v) phosphomolybdic acid solution containing 1% (w/v) ceric sulphate and 4% (v/v) H_2_SO_4_, followed by heating. NMR spectra were recorded on Bruker Avance spectrometers; for ^1^H (500 MHz), δ values were referenced to CDCl_3 _(CHCl_3 _at 7.24 ppm) and for ^13^C NMR (125.8 MHz) referenced to CDCl_3 _(77.23 ppm).

Ethyl acetate extracts of commercial serum (180 mL serum, 500 mg extract) was subjected to reverse phase flash column chromatography (FCC) with a step gradient elution; acetonitrile - water 25:75 to 100% acetonitrile. The fractions collected were analysed by LC/MS and MS/MS. The fractions containing the CRC biomarkers were pooled (12.5 mg). This procedure was repeated several times to obtain about 60 mg of CRC biomarker rich fraction. This combined sample was then subjected to FCC with a step gradient elution; hexane-chloroform-methanol and the fractions collected subjected to LC/MS and MS/MS analysis. The biomarker rich fraction labelled sample A (5.4 mg, about 65%) was analysed by NMR. Sample A (3 mg) was then treated with excess ethereal diazomethane and kept overnight at room temperature. After the removal of solvent, the sample was analyzed by NMR.

### TQ-MRM methodology

Serum samples were extracted as described for non-targeted FTICR-MS analysis, with the addition of 10 ug/mL [^13^C_1_]cholic acid to the serum prior to extraction (resulting in a final ethyl acetate concentration of [^13^C_1_]cholic acid of 36 nM. The ethyl acetate organic fraction was used for the analysis of each sample. A series of [^13^C_1_]cholic acid dilutions in ethyl acetate from Randox serum extracts was used to generate a standard curve ranging between 0.00022 μg/mL and 0.222 μg/mL. 100 μL of sample were injected by flow-injection analysis into the 4000QTRAP™ equipped with a TurboV™ source with an APCI probe. The carrier solvent was 90% methanol:10% ethyl acetate, with a flow rate of 360 μL/min into the APCI source. The source gas parameters were as follows: CUR: 10.0, CAD: 6, NC: -3.0, TEM: 400, GS1: 15, interface heater on. 'Compound' settings were as follows: entrance potential (EP): -10, and collision cell exit potential (CXP): -20.0. The method is based on the MRM of one parent ion transition for each of the C28 molecules (445.3-383.4 Da, 447.4-385.4 Da, and 449.4-405.4 Da) and a single transition for the internal standard (408.3-343.4 Da). Each of the transitions was monitored for 250 ms for a total cycle time of 2.3 s. The total acquisition time per sample was approximately 1 min. All accepted analyses showed R^2 ^correlation coefficients for the linear regression equation of >0.98. [^13^C_1_]cholic acid equivalents for each of the three C28 molecules were calculated by determining the percent recovery of [^13^C_1_]cholic acid in each sample by dividing the extrapolated concentration by 0.0148 ug/ml (36 nM, the theoretical amount present in the ethyl acetate extract of each sample). Metabolite concentrations represented as [^13^C_1_]cholic acid equivalents were then extrapolated, normalized by dividing by the percent recovery and multiplied by appropriate extraction dilution factors to yield a final serum concentration.

### Statistical analysis

FTICR-MS accurate mass array alignments were performed using *DISCOVA*metrics™ version 3.0 (Phenomenome Discoveries Inc, Saskatoon, Canada). Statistical analysis and graphs of FTICR-MS data was carried out using Microsoft Office Excel 2007 and distribution analysis of TQ-MRM data and was analysed using JMP version 8.0.1. Meta Analysis (Fisher's inverse chi-square method) was carried out using SAS 9.2 and R 2.9.0. Two-tailed unpaired Student's *t*-tests were used for determination of significance between CRC and controls. *P*-values of less than 0.05 were considered significant. Receiver operating curve (ROC) curves were generated using the continuous data mode of JROCFIT http://www.jrocfit.org.

## Results

### FTICR metabolomic profiling

**Figure 1:**
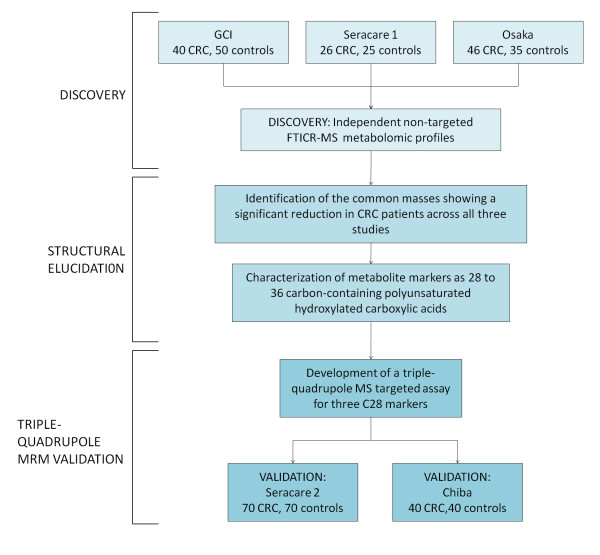
**Study design**. The study comprised three phases: Fourier transform ion cyclotron resonance mass spectrometry metabolomic discovery in three independent sample sets, structural investigation and determination of metabolic biomarkers as hydroxylated polyunsaturated ultra long-chain fatty acids and validation using a triple-quadrupole multiple reaction monitoring targeted assay.

**Figure 2:**
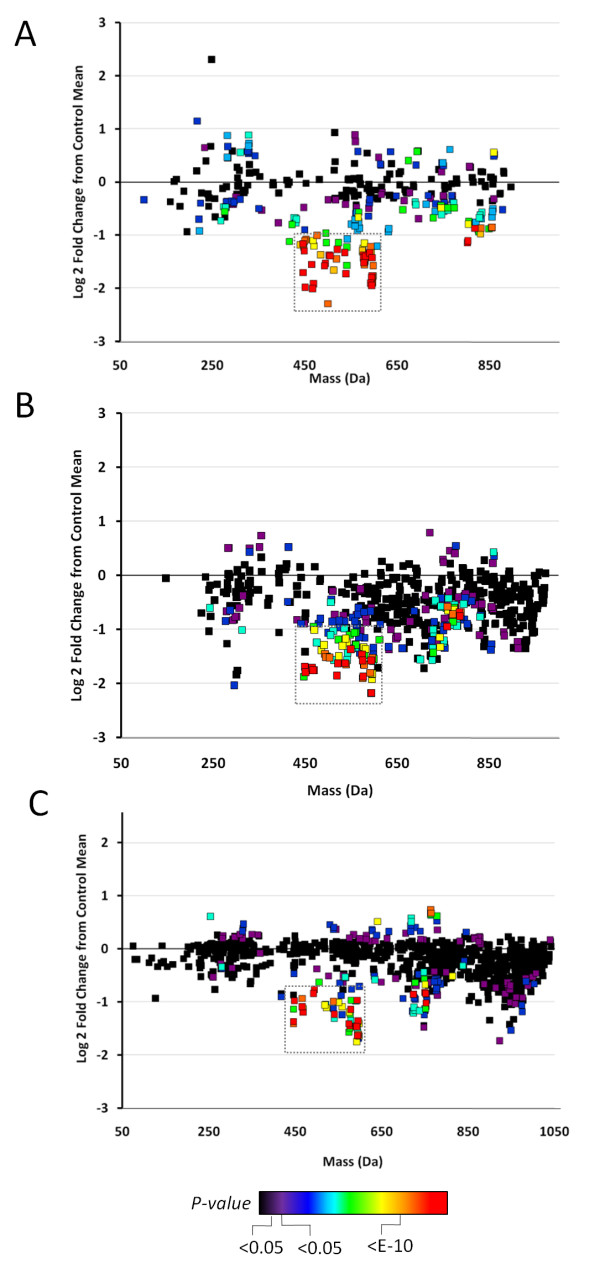
**Scatter plots of average sample peak intensity fold change between colorectal cancer (CRC) and normal patient sera in three independent studies**. Sample-specific peaks for all subjects were log2 normalized to the mean of the control population, and plotted according to mass (Da). Points are coloured according to significance based on an unpaired Students't-test (see legend). (A) Genomics Collaborative Inc discovery population, (B) Seracare 1 discovery population, (C) Osaka discovery population. The region boxed in grey represents the cluster of masses between 440 and 600 Da consistently reduced in the CRC patient population compared to controls in all three cohorts.

The experimental workflow for the studies described in this paper is summarized in Figure 1. Non-targeted metabolomic profiles of sera from three independent populations of treatment-naive CRC patients and geographically and ethnically matched healthy controls (summarized in Table 1) were generated over a 24-month period (that is, each study was separated by approximately 12 months). The first study comprised 40 CRC patients and 50 control subjects acquired from Genomics Collaborative, Inc (GCI); the second study comprised 26 CRC subjects and 25 controls acquired from Seracare Lifesciences Inc; and the third study included 46 CRC and 35 controls prospectively collected in Osaka, Japan. In all cases, serum metabolites were captured through a liquid extraction process (see Methods), followed by direct infusion of the extracts using negative electrospray ionization (nESI) and negative atmospheric pressure chemical ionization (nAPCI) on an FTICR mass spectrometer. The resulting spectral data of all the subjects for each study was aligned within 1 ppm mass accuracy, background peaks were subtracted, and a two-dimensional array table comprising the intensities each of the sample-specific spectral peaks was created using custom informatics software (see Methods). Metabolic differences between CRC patient and control profiles for the three independent studies were visualized by plotting the control mean-normalized log ratio peak intensities across the detected mass range as shown in Figures 2A to 2C. In each independent study, a region of spectra between approximately 440 and 600 Da showed peaks consistently reduced in intensity in CRC patients relative to controls (green, yellow, orange and red points in Figure 2). On average, this cluster of masses showed between 50% and 75% reduction in CRC patient serum compared to the respective controls, with p-values of 1 × 10^-5 ^or lower in each study.

The overlap between each of the discovery studies was further investigated by ranking the top 50 masses based upon *P*-value from each study and comparing them with masses showing a significant difference (*P *< 0.05 between CRC and controls) in the other studies as shown in Table 2. For example, 46 of the top 50 metabolites (92%) with the lowest p-values in the GCI discovery set were also significant (*P *< 0.05) in the Seracare 1 dataset, while 31 out of the 50 GCI masses were significant (*P *< 0.05) in the Osaka dataset. Likewise, the top 50 metabolites in the Osaka study showed 88% and 94% redundancy with metabolites showing *P *< 0.05 in the GCI and Seracare 1 studies, respectively. These results indicated a very high degree of commonality among significantly differentiated masses across the three studies, and in fact, 63% of the top 50 masses in each study were also present within the top 50 of at least one of the other two studies (see Additional File 2). Of the top 50 rank-ordered masses, only those identified in more than one study were found to exist within the 440 to 600 Da mass range highlighted above and there was not a single peak detected outside this region which was significantly different between CRCs and controls in any two of the studies. Filtering for metabolic differences detected exclusively in all three studies (as well as removal of C13 isotopic peaks and redundant masses detected in both ESI and APCI), resulted in 13 masses representing individual ^12^C metabolites as shown in Table 3. These represented the most statistically significant and robust discriminators among the three studies. Subsequent molecular formula assignments, as discussed further below, as well as related expression profiles, suggested that the metabolites belonged to a related chemical family.

**Table 2:** Percent overlap between top 50 most discriminating masses (based on student's *t*-test) of each discovery project and masses showing *P *< 0.05 in the remaining cohorts

	Genomics Collaborative (*P *< 0.05)	Seracare (*P *< 0.05)	Osaka (*P *< 0.05)
**GCI (Top 50)**	-	46 (92%)	31 (62%)
**Seracare 1 (Top 50)**	35 (70%)	-	27 (54%)
**Osaka (Top 50)**	44 (88%)	47 (94%)	-

**Figure 3:**
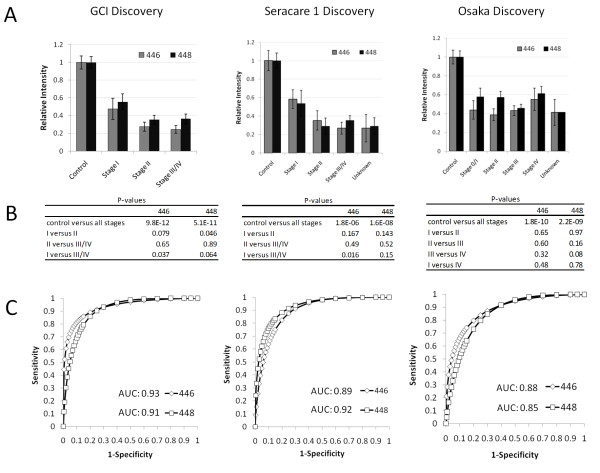
**Relative intensities of metabolites 446 and 448 by disease stage and the area under the curves for each discovery dataset**. (A) Bar charts of relative intensity versus disease stage in each sample set; (B) summary of *P*-value comparisons between disease stages and controls for metabolites 446 and 448; (C) receiver operating curve analysis based on markers 446 and 448 and all CRCs versus all controls in each discovery set.

The relative intensities of the two lowest molecular weight molecules with nominal masses of 446 and 448 are shown in Figure 3A. We observed little to no correlation between the reduction of the metabolites and disease stage (Figures 3A and 3B), and ROC curve analysis resulted in an average area under-the-curve (AUC) of 0.91 ± 0.03 (Figure 3C; individual AUCs shown) across all three studies for all stages combined.

Computational assignments of reasonable molecular formulas were then carried out for the 13 masses identified above, as well as the top 50 for each discovery set shown in Additional File 2. The assignments were based on a series of mathematical and chemometric rules as described previously [23], which are reliant on high mass accuracy for precise prediction. The algorithm computes the number of carbons, hydrogens, oxygens and other elements, based on their exact mass, which can be assigned to a detected accurate mass within defined constraints. Logical putative molecular formulas were computed for masses in Table 3 (and Additional File 2), resulting in elemental compositions containing either 28, 30, 32 or 36 carbons and four to six oxygen. We used this information in the subsequent section to select appropriate molecules for structural comparison studies. Collectively, the results indicated a consistent 50% to 75% reduction of organically soluble oxygenated metabolites ranging between 28 and 36 carbons in length, in the serum of CRC patients compared to controls.

**Table 3:** List of 13 masses detected among the top 50 masses inclusive to all three discovery projects

GCI						
**Rank order**	**Detected mass**	**Molecular formula**	**Part per million**	**Analysis mode**	***P *value**	**Ratio (CRC/normal)**

6	446.3406	C28H46O4	2.22	NAPCI	6.4E-13	0.31
13	448.3563	C28H48O4	2.32	NAPCI	2.5E-12	0.41
8	466.3661	C28H50O5	0.59	NAPCI	9.4E-13	0.25
7	468.3840	C28H52O5	5.39	NAPCI	9.0E-13	0.27
21	492.3829	C30H52O5	2.89	NAPCI	8.5E-11	0.33
24	494.3977	C30H54O5	1.16	NAPCI	1.9E-10	0.35
29	518.3976	C32H54O5	0.92	NAPCI	1.6E-09	0.37
12	538.4259	C32H58O6	4.76	NAPCI	2.5E-12	0.30
44	574.4607	C36H62O5	1.7	NAPCI	1.6E-08	0.40
26	576.4771	C36H64O5	2.99	NAPCI	3.0E-10	0.37
32	578.4931	C36H66O5	3.59	NAPCI	3.2E-09	0.34
11	592.4711	C36H64O6	1.37	NAPCI	2.2E-12	0.27
15	594.4851	C36H66O6	1.41	NAPCI	6.3E-12	0.26

**Seracare**						

45	446.3413	C28H46O4	3.79	NAPCI	1.8E-06	0.36
9	448.3570	C28H48O4	3.88	NAPCI	1.6E-08	0.36
3	466.3664	C28H50O5	1.23	NAPCI	8.5E-10	0.34
6	468.3847	C28H52O5	6.89	NAPCI	4.9E-09	0.36
17	492.3835	C30H52O5	4.11	NAPCI	4.6E-08	0.42
34	494.3971	C30H54O5	0.05	NAPCI	6.6E-07	0.41
11	518.3968	C32H54O5	0.63	NAPCI	2.2E-08	0.33
18	538.4263	C32H58O6	5.5	NAPCI	7.8E-08	0.38
32	574.4595	C36H62O5	0.39	NAPCI	6.1E-07	0.32
42	576.4768	C36H64O5	2.47	NAPCI	1.0E-06	0.37
49	578.4933	C36H66O5	3.93	NAPCI	3.2E-06	0.42
30	592.4721	C36H64O6	3.06	NAPCI	5.6E-07	0.27
50	594.4851	C36H66O6	1.41	NAPCI	3.7E-06	0.32

**Osaka**						

6	446.3400	C28H46O4	0.87	NESI	1.8E-10	0.44
13	448.3556	C28H48O4	0.76	NESI	2.2E-09	0.54
1	466.3663	C28H50O5	1.02	NESI	2.9E-12	0.50
5	468.3815	C28H52O5	0.05	NESI	1.8E-10	0.49
4	492.3814	C30H52O5	0.15	NESI	7.1E-11	0.57
23	494.3969	C30H54O5	0.45	NESI	2.0E-07	0.62
39	518.3975	C32H54O5	0.72	NAPCI	5.8E-06	0.52
19	538.4237	C32H58O6	0.67	NESI	4.7E-08	0.58
16	574.4600	C36H62O5	0.48	NESI	3.8E-09	0.42
7	576.4756	C36H64O5	0.39	NESI	3.0E-10	0.42
14	578.4910	C36H66O5	0.04	NESI	2.6E-09	0.50
15	592.4703	C36H64O6	0.02	NESI	3.3E-09	0.41
3	594.4859	C36H66O6	0.07	NESI	6.8E-11	0.40

Indicated are the rank order based on *P*-value, detected accurate mass, the computationally predicted molecular formula, the mass difference between the detected mass and mass of the predicted molecular formula in part per million, the mode of analysis (electrospray ionization, ESI; atmospheric pressure chemical ionization, APCI), the *P*-value (based on an unpaired student's *t*-test) between the average peak intensity of control subjects versus colorectal cancer (CRC) patients, and the average peak intensity ratio between CRC patients and controls.

### Structural elucidation

**Figure 4:**
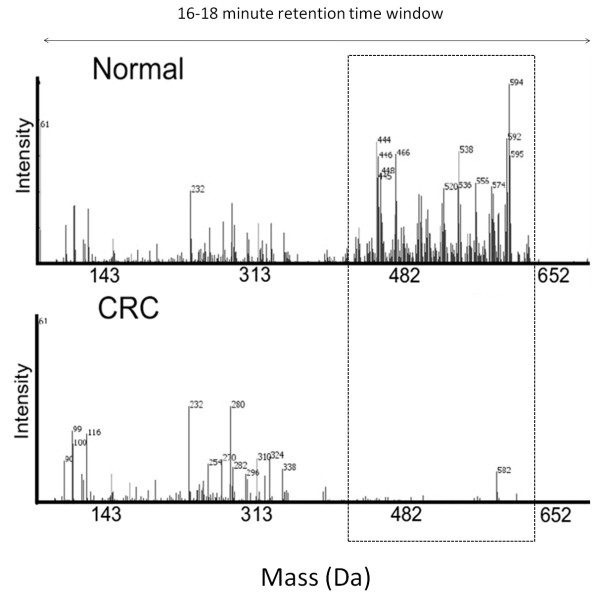
**Extracted mass spectrum of serum from normal subjects and colorectal cancer (CRC) patients**. Extracts from five representative CRC and five control samples from the Genomics Collaborative discovery set were subject to high performance liquid chromatography followed by full-scan detection on an Applied Biosystems QSTAR XL™ mass spectrometer in atmospheric pressure chemical ionization negative mode. The average intensities of all ions within the mass range 100 to 700 Da eluting between 16 and 18 min are shown for each cohort. The boxed region indicates spectral features present in normal patients but absent from CRC-positive serum.

Selected ethyl acetate extracts of serum from the GCI cohort used in the FTICR-MS work described above were re-analysed using HPLC coupled to a quadrupole time-of-flight (Q-TOF) MS in full-scan APCI negative ion mode. Consistent with the FTICR-MS results, a cluster of peaks between approximately 440 and 600 Da at a retention time of between 16 and 18 min following reverse-phase HPLC was detected in asymptomatic control sera, but was absent from CRC patient serum (Figure 4). Molecular ions from all six C28 biomarkers (m/z 446, m/z 448, m/z 450, m/z 464, m/z 466 and m/z 468) as well as many of the remaining C32 and C36 markers were easily detectable within normal serum. Extracted masses up to 400 Da within the 16-18 min retention time showed similar peak intensities in both populations (Figure 4, region to the right of the box), as did extracted mass spectra at other retention times (not shown), reinforcing the specificity of this depleted metabolic region for CRC patient serum.

Tandem mass spectrometric fragmentation fingerprints were next generated for the six C28 biomarkers (Table 4, see Additional Files 3,4,5,6,7,8) and for the higher C32 and C36 biomarkers (see Additional File 9). The MS/MS and MS3 fragmentation data of the six C28 biomarkers were dominated by peaks resulting from losses of H_2_O (m/z 427, 429, 431, 445, 447 and 449), losses of two molecules of H_2_O (m/z 409, 411, 413, 427, 429, 431), losses of CO_2 _(m/z 401, 403, 405, 419, 421, 423) and losses of CO_2 _and H_2_O (m/z 383, 385, 387, 401, 403, 405), indicating the presence of carboxylic acid functionality and two or more hydroxyl groups. The molecular formulae, organic properties of the molecules and the tandem MS data suggested that the metabolites were derivatives or analogues of one or more possible classes of molecules including fat soluble vitamins such as retinol and retinoic acid (vitamin A), calciferols (vitamin D), tocopherols (vitamin E), phylloquinones (vitamin K), steroids or bile acids, or long chain polyunsaturated hydroxy fatty acids. Tandem mass spectrometric fragmentation fingerprints of standards for each of these metabolic classes were therefore generated including 5S,6S-(7E,9E,11Z,14Z)-dihydroxyeicosatetraenoic acid (**1**), 15S-Hydroxy-(5Z,8Z,11Z,13E)-eicosatetraenoic acid (**2**) and 8R-Hydroxy-(5Z,9E,11Z,14Z)-eicosatetraenoic acid (**3**), α-tocopherol (**4**) γ-tocopherol (**5**), 13-(6-hydroxy-2,7,8-trimethylchroman-2-yl)-2,6,10-trimethyltridecanoic acid (**6**), 16-(4,5-dimethyl-3,6-dioxo cyclohexa-1,4-dienyl)-2,6,10,14-tetramethylhexadecanoic acid (**7**), 6-hydroxy-2,7-dimethyl-2-(4,8,12-trimethyltridecyl)chroman-8-carbaldehyde (**8**), 6-hydroxy-2,7-dimethyl-2-(4,8,12-trimethyltridecyl)chroman-8-carboxylic acid (**9**), calciferol (**10**), cholecalciferol (**11**), ergosterol (**12**), phylloquinone (**13**), retinol (**14**) and 3β,7α-dihydroxy-5-cholestenoic acid (**15**) (Table 5). The resulting MS/MS data for vitamins A, D, E, K as well as the steroidal molecules (**4 **- **15**) showed no similarity to any of the metabolomic biomarkers; for vitamin E type molecules, all had diagnostic fragments characteristic of their chroman rings (m/z 163, 149, 149, 149, 163 and 179 for **4**, **5, 6, 7, 8 **and **9 **respectively), for vitamin D and analogues, diagnostic fragments formed as a result of the loss of the side chain (m/z 271, 273 and 253, for **10**, **11 **and **13, **respectively), for phylloquinone (**13**), the diagnostic fragment m/z 187 for the quinone ring system was prominent, for vitamin A (**14**), the fragment m/z 269 (M + H - H_2_O) loses the cyclohexyl ring moiety to form a diagnostic m/z 145 for retinol and for 3β,7α-dihydroxy-5-cholestenoic acid (**15) **the diagnostic retro diels alder fragment at m/z 277 was observed. In addition to this, other carboxylic acid standards with a pregnane ring system, as in **15 **(for example, chenodeoxycholic acid and cholic acid), do not show losses of CO_2 _upon MS/MS fragmentation (not shown). However, MS/MS fragmentation data of hydroxy fatty acid standards **1**, **2 **and **3 **(Table 5) showed peripheral cut ions similar to those produced by MS/MS of the CRC biomarkers, and consistent with what has been described by others for various hydroxylated long-chain fatty acids [29-33]. For example, marker m/z 446 showed peripheral cut ions 427 [M - H - H_2_O]^-^, 401 [M - H - CO_2_]^-^, 409 [M - H - 2H_2_O]^-^, 383 [M - H - CO_2 _- H_2_O]^- ^and 365 [M - H - CO_2 _- 2H_2_O]^- ^and chain cut ions, 223, 205, 277 as well as others (see Table 5 and Additional File 3). Similar ions were obtained for the other C28, C32 and C36 metabolites (Table 4 and Additional File 9). Collectively, these deductions indicated that the metabolomic markers were not analogues of vitamins A, D, E, K and steroids, but rather long-chain hydroxy fatty acids containing varying degrees of unsaturation. We collectively refer to these metabolites as hydroxylated polyunsaturated ultra long-chain fatty acids (hPULCFAs; where the term 'ultra' has been used to refer to C30 and longer chain fatty acids [34]).

**Table 4:** Tandem-mass spectrometry (MS) analysis of selected 28-carbon containing masses

Marker nominal neutral mass		446	448	450	464	466	468
**[M-H]-(%)**		**445 **(100%)	**447 **(52%)	**449 **(92%)	**463 **(70%)	**465 **(100%)	**467 **(100%)

**Chain cut ions (%)**		**223 **(18%)	**277 **(11%)	**171 **(7%)	**277 **(10%)	**241 **(7%)	**187 **(12%)
		**222 **(11%)	**239 **(5%)	**127 **(9%)	**241 **(68%)	**223 **(3%)	**169 **(3%)
		**207 **(3%)	**207 **(3%)	**125 **(12%)	**223 **(15%)	**215 **(2%)	**141 **(2%)
		**205 **(11%)	**169 **(6%)	**113 **(38%)	**185 **(8%)	**185 **(4%)	**113 **(4%)
		**113 **(5%)	**113 **(25%)		**167 **(4%)	**167 **(4%)	
					**113 **(28%)	**113 **(7%)	

Peripheral cut ions (%)	**Loss of H**_2_**O**	**427 **(50%)	**429 **(35%)	**431 **(80%)	**445 **(46%)	**447 **(45%)	**449 **(84%)
	**Loss of 2H**_2_**O**	**409 **(8%)	**411 **(6%)	**413 **(13%)	**427 **(6%)	**429 **(8%)	**431 **(10%)
	**Loss of CO**_2_	**401 **(95%)	**403 **(100%)	**405 **(100%)	**419 **(100%)	**421 **(45%)	**423 **(25%)
	**Loss of CO**_2_**and H**_2_**O**	**383 **(28%)	**385 **(15%)	**387 **(32%)	**401 **(24%)	**403 **(20%)	**405 **(13%)
	*Loss of CO_2_**and 2H**_2_**O**	**365**	**367**	**369**	**383 **(2%)	**385 **(4%)	**387 **(3%)
	*Loss of 3H_2_**O**				**409**	**411**	**413**

**Secondary daughter ions (%)**		**357 **(5%)	**331 **(3%)	**307 **(5%)	**347 **(5%)	**349 **(4%)	**349 **(1%)
		**329 **(11%)	**305 **(3%)	**291 **(7%)	**319 **(5%)	**321 **(2%)	**323 **(2%)
		**261 **(3%)	**359 **(2%)	**295 **(5%)	**295 **(6%)	**297 **(3%)	**309 **(2%)
		**241 **(3%)	**289 **(3%)	**281 **(5%)	**281 **(5%)	**281 **(3%)	**297 **(6%)
		**233 **(5%)	**245 **(3%)	**279 **(9%)	**279 **(5%)	**279 **(15%)	**281 **(3%)
		**207 **(11%)	**125 **(6%)	**263 **(7%)	**267 **(5%)	**261 **(3%)	**279 **(5%)
		**177 **(11%)	**123 **(3%)	**261 **(5%)	**249 **(6%)	**251 **(3%)	**269 **(5%)
		**123 **(5%)	**121 **(3%)	**169 **(5%)	**195 **(10%)	**195 **(2%)	**263 **(8%)
		**109 **(11%)	**111 **(5%)	**111 **(5%)	**141 **(1%)	**141 **(2%)	**251 **(4%)
		**97 **(16%)	**97 **(5%)	**97 **(8%)	**127 **(9%)	**123 **(4%)	**243 **(2%)
		**83 **(11%)	**59 **(3%)	**83 **(5%)	**121 **(6%)	**113 **(5%)	**215 **(4%)
		**59 **(11%)		**59 **(1%)	**101 **(6%)	**101 **(3%)	**213 **(3%)
					**97 **(4%)	**97 **(32%)	**197 **(3%)
					**83 **(2%)	**83 **(2%)	**125 **(4%)
					**59 **(2%)	**59 **(2%)	**111 **(3%)
							**98 **(2%)
							**57 **(1%)

*Ions m ay have been obtained from MS^3 ^experiments

**Table 5:** Tandem mass spectrometric results of various standards

Standard		1	2	3	4	5	6	7	8	9	10	11	12	13	14	15
**[M-H]-(%)**		**335 **(45%)	**319 **(100%)	**319 **(100%)	**429 **(14%)	**415 **(28%)	**445 **(90%)	**445 **(68%)	**429 **(100%)	**445 **(64%)	**397 **(6%)	**385 **(2%)	**397 **(5%)	**451 **(28%)	**287 **(1%)	**431 **(100%)

*Chain cut ions (%)		**219 **(15%)	**219 **(80%)	**203 **(3%)												
		**201 **(1%)	**203 **(15%)	**163 **(25%)												
		**115 **(100%)	**175 **(55%)	**155 **(60%)												
			**113 **(20%)	**127 **(10%)												
				**111 **(5%)												

*Peripheral cut ions (%)	**Loss of H**_2_**O**	**317 **(16%)	**301 **(60%)	**301 **(55%)			**427 **(50%)		**401 **(30%)	**401 **(28%)	**379 **(10%)	**367 **(18%)	**379 **(5%)		**269 **(1%)	**413 **(1%)
	**Loss of 2H**_2_**O**	**299**														
	**Loss of CO**_2_	**291 **(2%)	**275 **(20%)	**275 **(4%)			**401 **(1%)	**401 **(35%)								
	**Loss of CO**_2_**and H**_2_**O**	**273 **(6%)	**257 **(70%)	**257 **(80%)												
	*Loss of CO_2_**and 2H**_2_**O**															
	*Loss of 3H_2_**O**															

**Secondary daughter ions (%)**		**189 **(1%)	**167 **(5%)	**291 **(1%)	**414 **(5%)	**400 **(78%)	**295 **(70%)	**386 **(52%)	**163 **(100%)	**386 **(43%)	**309 **(5%)	**273 **(3%)	**295 **(5%)	**436 **(10%)	**187 **(5%)	**399 **(100%)
		**163 **(1%)	**149 **(2%)	**171 **(1%)	**163 **(100%)	**175 **(13%)	**149 **(90%)	**179 **(60%)	**135 **(20%)	**179 **(50%)	**213 **(15%)	**259 **(25%)	**253 **(6%)	**241 **(21%)	**173 **(10%)	**393 **(40%)
		**145 **(2%)	**121 **(20%)	**107 **(1%)	**135 **(8%)	**149 **(100%)	**136 **(100%)	**135 **(100%)	**218 **(5%)	**166 **(20%)	**201 **(30%)	**255 **(15%)	**211 **(12%)	**227 **(62%)	**159 **(18%)	**373 **(40%)
		**99 **(1%)	**99 **(1%)	**59 **(1%)		**121 **(90%)	**121 **(20%)	**107 **(25%)	**123 **(5%)	**135 **(100%)	**173 **(37%)	**213 **(20%)	**159 **(20%)	**223 **(48%)	**145 **(28%)	**355 **(40%)
		**95 **(1%)	**59 **(1%)							**122 **(25%)	**159 **(35%)	**173 **(47%)	**161 **(15%)	**213 **(55%)	**119 **(15%)	**337 **(20%)
		**71 **(1%)								**107 **(25%)	**107 **(90%)	**161 **(52%)	**147 **(20%)	**199 **(57%)	**105 **(20%)	**223 **(40%)
		**59 **(1%)									**81 **(68%)	**159 **(75%)	**107 **(25%)	**187 **(100%)	**95 **(36%)	**85 **(40%)
											**69 **(100%)	**149 **(40%)	**105 **(15%)	**185 **(52%)	**93 **(28%)	
												**147 **(72%)	**95 **(38%)	**171 **(55%)	**81 **(58%)	
												**107 **(100%)	**93 **(17%)	**71 **(86%)	**69 **(100%)	
												**81 **(82%)	**83 **(25%)			
													**81 **(40%)			
													**36 **(100%)			

*This terminology is specific to fatty acid fragmentation.

5S,6S-(7E,9E,11Z,14Z)-dihydroxyeicosatetraenoic acid (1), 15S-Hydroxy-(5Z,8Z,11Z,13E)-eicosatetraenoic acid (2) and 8R-Hydroxy-(5Z,9E,11Z,14Z)-eicosatetraenoic acid (3) (Table 6), α-tocopherol (4) γ-tocopherol (5), 13-(6-hydroxy-2,7,8-trimethylchroman-2-yl)-2,6,10-trimethyltridecanoic acid (6), 16-(4,5-dimethyl-3,6-dioxo cyclohexa-1,4-dienyl)-2,6,10,14-tetramethylhexadecanoic acid (7), 6-hydroxy-2,7-dimethyl-2-(4,8,12-trimethyltridecyl)chroman-8-carbaldehyde (8), 6-hydroxy-2,7-dimethyl-2-(4,8,12-trimethyltridecyl)chroman-8-carboxylic acid (9), calciferol (10), cholecalciferol (11), ergosterol (12), phylloquinone (13), retinol (14) and 3β,7α-dihydroxy-5-cholestenoic acid (15).

Next, an enrichment strategy using bulk serum extracts and a two-stage flash column chromatography approach followed by nuclear magnetic resonance (NMR) analysis was carried out to provide further structural verification of the hPULCFAs. First, reverse phase FCC using a water-acetonitrile solvent gradient was performed and the resulting fractions analysed by LC/MS. Fractions containing the hPULCFAs (fraction 9, Additional Files 10 and 11) were pooled and subjected to normal phase FCC using chloroform-methanol mixtures to obtain an approximately 65% rich semi-purified fraction labelled sample A (Additional File 12). LC and MS/MS analyses (MS2 and MS3) data on sample A were used to track and confirm enrichment of the markers. NMR (^1^H, ^13^C and 2D) analyses on sample A and its methyl esters revealed resonances and correlations (Table 6) consistent with very long chain polyunsaturated hydroxy fatty acids with observance of some suppression of resonances for hydrogen atoms attached to sp^2 ^carbons.

**Table 6:** ^1^H nuclear magnetic resonance (NMR) data of colorectal cancer (CRC) biomarker pool (sample A) and their methyl esters

Types of protons	CRC biomarker pool	Methyl esters of CRC biomarker pool
*CH*_3_	0.83-0.90	0.83-0.90
*CH*_2_	1.21-1.24, m	1.21-1.24, m
-*CH*_2_CH_2_COOH	1.57-1.65, m	1.53-1.69, m
-*CH*_2_CH=CH-	1.98-2.08, m	1.94-2.03, m
*CH*_2_COO	2.23-2.28, m	2.23-2.31, m
-CH=CH-*CH*_2_-CH=	2.75-2.79, m	2.74-2.82, m
*OCH*_3_	-	3.64, s
-*CH*(OH)CH=	3.45-3.71, 4.03-4.26	4.02-4.12, 4.16-4.26, 4.58-4.60
-*CH*=	5.10-5.47, m	5.08-5.40, m
-CH(OH)*CH*=	5.76-5.91, m	5.75-5.90, m

*NMR solvent is CDCl_3_, signals assigned using 2D NMR experiments (HMQC and HMBC)

### Independent validation using MRM methodology

**Figure 5:**
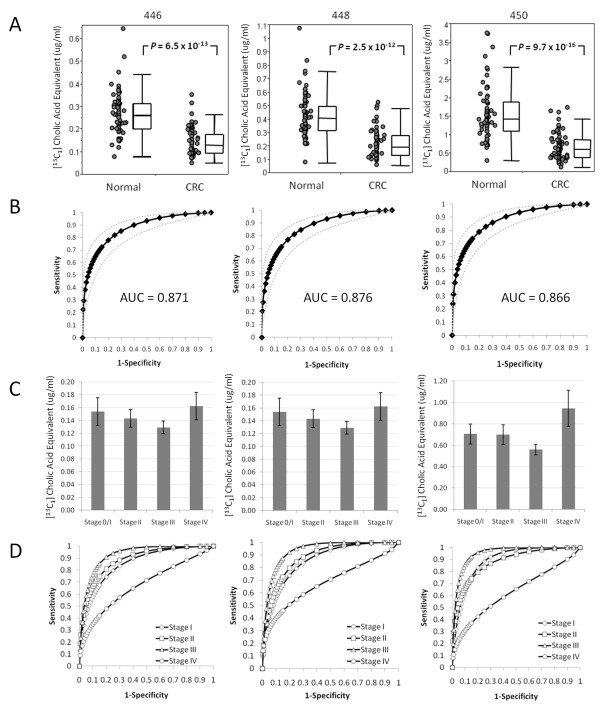
**Results of triple-quadrupole multiple reaction monitoring analysis of the Seracare 2 validation sample set**. (A) Scatter plots of the concentrations of hydroxylated polyunsaturated ultra long chain fatty acids (hPULCFAs) 446, 448 and 450 expressed as [^13^C_1_]-cholic acid equivalents in asymptomatic normal controls and pre-treatment colorectal cancer patients, (B) receiver operating curve (ROC) analysis based upon the corresponding scatter plots in (A). Grey dotted lines indicate the 95% confidence interval. (C) Bar charts of the average concentration equivalents of hPULCFAs by disease stage. Error bars represent standard errors of the mean. (D) ROC analysis by disease stage.

Reduced levels of hPULCFAs in the blood of CRC patients was further confirmed using a MS/MS approach (see methods) in two more independent populations. The approach is based upon the measurement of parent-daughter fragment ion combinations (referred to as MRM) for quantifying analytes [28,35]. We developed an assay to specifically measure semi-quantitatively three of the 28 carbon hPULCFAs with four oxygens (parent masses 446, 448 and 450; C28H46O4, C28H48O4 and C28H50O4, respectively) as described in the methods. The first study comprised 70 treatment-naive CRC subjects and 70 matched controls, all of which were Caucasians from the USA. The [^13^C_1_]cholic acid equivalent concentrations of the three 28-carbon hPULCFAs (named according to nominal mass 446, 448 and 450) for each subject are shown in Figure 5A. Significantly lower levels (*P *< 0.001, actual values shown in Figure 5A) of each of the metabolites was observed in treatment-naive CRC-positive subjects compared to controls. ROC analysis resulted in AUCs of 0.87 ± 0.005 for each of the 28-carbon containing hPULCFAs (Figure 5B). Plotting patients by disease stage showed a slight (but not significant) reduction between stage I and III, with stage IV subjects showing the least reduction (Figures 5C and 5D), albeit it only seven subjects. The corresponding average AUCs of the 28-carbon pool by stage were 0.87 for stage I, 0.88 for stage II, 0.94 for stage III and 0.66 for stage IV.

**Figure 6:**
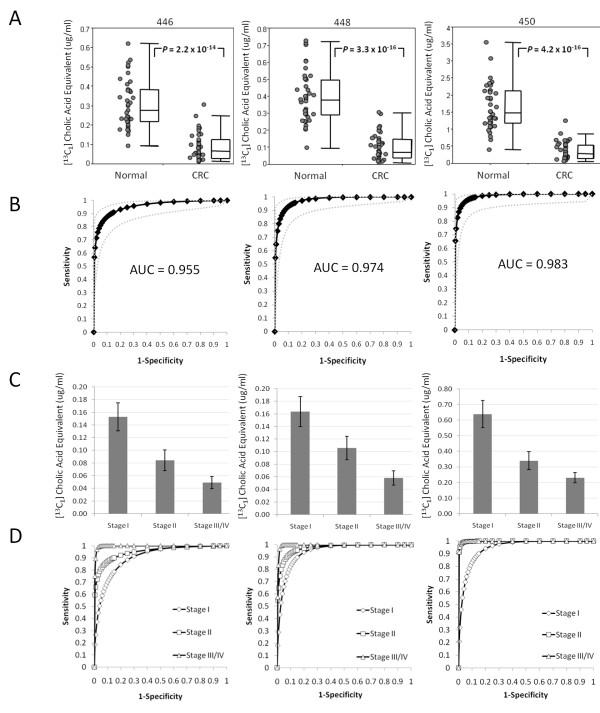
**Results of triple-quadrupole multiple reaction monitoring analysis of the Chiba validation sample set**. (A) Scatter plots of the concentrations of hydroxylated polyunsaturated ultra long-chain fatty acids (hPULCFAs) 446, 448 and 450 expressed as [^13^C_1_]-cholic acid equivalents in asymptomatic normal controls, and pre-treatment colorectal cancer patients, (B) receiver operating curve (ROC) analysis based upon the corresponding scatter plots in (A). Grey dotted lines indicate the 95% confidence interval. (C) Bar charts of the average concentration equivalents of hPULCFAs by disease stage. Error bars represent standard errors of the mean. (D) ROC analysis by disease stage.

We next used the MRM method to characterize another independent population of CRC and control subjects from Chiba, Japan. Serum from 40 pre-treatment CRC subjects and 40 controls were analysed and a significant reduction was again observed in the CRC-positive group (Figure 6A). The corresponding average AUC for the three metabolites was 0.97 ± 0.014 (Figure 6B). In this study, a significant correlation with stage was observed (*P *< 0.05) for all comparisons between stages I, II and III/IV (Figures 6C and 6D). The AUCs by stage were 0.93 for stage I, 0.97 for stage II and 1.0 for stage III/IV (two stage IVs were grouped with stage III; Figure 6D).

## Discussion

We report here on the discovery of novel hydroxylated polyunsaturated ultra long-chain fatty acids containing between 28 and 36 carbons reduced in the serum of CRC patients compared to healthy asymptomatic controls. The utility of non-targeted metabolomics using high resolution FTICR-MS coupled with flow injection technology for biomarker discovery was demonstrated by applying the technology to three independent test populations. In contrast to the 'training/test-set' approach often used by splitting a single sample set in half to validate the performance of biomarkers [36-38], which often relies on complex algorithms (see review [39]) and can result in bias [40], we carried out fully independent discovery analyses on three separate sample sets of matched cases and controls of different ethnic backgrounds collected from multiple sites around the world to ensure a high degree of robustness and minimal chance of sampling bias. Of the top 50 metabolic discriminators discovered in the Osaka set, 44 and 47 of these were also significantly changed in the GCI and Seracare sets, respectively. This remarkable inter-study agreement indicates that not only is non-targeted FTICR-MS technology a reproducible biomarker discovery engine, but that disease-related metabolomic changes can be highly conserved across geographic locations and races. The reduction of hPULCFAs in the serum of CRC patients was further validated by translation of the non-targeted FTICR-MS discovery into a simple targeted TQ-MRM method for three hPULCFAs, which was used on two further independent and ethnically diverse case-control test populations. ROC AUCs generated from the TQ-MRM method on the two validation studies were consistent with those based upon the same fatty acids detected in the three FTICR-MS discovery studies (Figures 3, 5 and 6). In total, five independent study populations collectively comprising 222 treatment-naive CRC patient samples and 220 disease-free asymptomatic controls were evaluated using two different analytical methods. Indeed, the likelihood of the reported association between the reduction of hPULCFAs and CRC being a false positive result across the five independent sets of samples is astronomically low. Meta-analysis was performed on the false positive rates using *Fisher's Inverse Chi-square Method *(; *p *= *P*-values of five independent samples, *k *= five different samples, *C *= upper tail of the chi-square distribution with 2 k degrees of freedom ( = 18.31))[41,42]. Based upon the meta-analysis, the resulting *P*-values for markers 446 and 448 were more significant than the individual *P*-values, at 2.96 × 10^-47 ^and 8.11 × 10^-49^, respectively. Although there were differences in the median ages between the CRC and control cohorts in two of the studies, there was no statistically significant trend between age and hPULCFA levels within the individual cohorts and we observed no significant difference between hPULCFA concentrations among the controls from the different populations (not shown). We also observed no differences between genders, and although there were slightly higher BMI levels in the control cohorts for the GCI and Seracare 1 cohorts, the BMIs were matched in the second Seracare validation population suggesting the markers are not related to BMI. A prospective analysis of disease-free subjects equally distributed across various age groups is underway specifically to address any potential age or BMI effects in more detail. Overall our results indicate with a high degree of confidence that a reduction in these metabolites is correlated with the presence of CRC.

The FTICR-MS provided resolution sufficient for confident molecular formula predictions based upon accurate mass in conjunction with extraction, ionization, and statistical correlative information. Although multiple elemental compositions were theoretically assignable to given biomarker masses, only formulas having 28 to 32 carbons, and four to six oxygen were consistently assignable to common masses detected in two or three of the discovery sets. Given a high degree of statistical interaction between the sample-to-sample expression profiles of the hPULCFAs (that is, a high degree of correlation between the relative intensities of the markers across subjects) we suspected they were all part of the same metabolic system and should therefore show related compositions. Detection in negative ionization mode also reduced the likelihood that nitrogen was present in any of the compositions. This information in conjunction with tandem mass spectrometry showing prominent losses of water and carbon dioxide enabled the determination of molecular formulas as shown in Table 3 and Additional File 2. A number of candidate classes of molecules theoretically fitting the molecular formula class were easily excluded using tandem MS. For example, we observed no fragments indicative of condensed ring systems such as those in steroids or vitamin D, and no fragments indicative of chroman ring systems such as those observed in the vitamin E tocopherols. Several other classes of molecules including vitamin K and retinol, and bile acids such as cholic acid and 3β,7α-dihydroxy-5-cholestenoic acid also did not show comparable fragmentation patterns. However, the similarity in fragmentation pattern, particularly in the relative abundances of daughter ions resulting from losses of CO_2 _and H_2_O, and chain cut ions from the hPULCFAs to known hydroxy fatty acid standards as well as other fatty acids reported in the literature such as the resolvins and protectins (discussed below), allowed for the identification of the metabolites as hydroxylated polyunsaturated ultra long-chain fatty acids. Examination of the MS/MS data for the C28 series (masses 446, 448, 450, 464, 466 and 448) revealed a consistent 113 Da daughter ion, which we conjecture to represent the carboxy-terminus chain fragment -CH_2_-CH=CH-CH_2_-CH_2_-COOH. In addition, a consistent loss of 54 (-CH=CH-CH_2_-CH_2_-) from the [M-(CO_2_**+**H_2_O)] daughter ion was observed for the 446, 448, 464 and 466, but not the 450 and 468 molecules, suggesting that (1) the 450 and 468 may have a saturated carboxy terminal region and (2), that there are likely no hydroxyl moieties within this region of the molecule. MS/MS data of all the C28 and other markers also did not show the diagnostic fragment obtained with a 1,2-diol motif as observed for **1 **(base peak is chain cut ion at m/z 115) and NMR on fractions enriched via flash-column chromatography showed lower than expected integration values obtained for the ^1^H NMR signals at δ 2.78 (methylene interruptions between double bond carbons) and at δ 5.12 - 5.90 (hydrogen atoms on double bond carbons). Cumulatively these results suggested that the hydroxyl groups in the molecules are likely bonded to the carbon atoms between the sp^2 ^carbons at least seven carbons from the carboxy end. Confirmation of the exact positions of the hydroxyl groups and precise locations of unsaturations in individual hPULCFAs using preparatory HPLC and chemical synthesis is in progress and will be reported in subsequent publications.

Interestingly, the metabolite markers reported in this study represent a human-specific metabolic system. We analysed serum samples from multiple species, including rat, mouse and bovine, as well as multiple different sample sources including numerous cell lines, conditioned media, tumour and normal colonic tissue from patients in the GCI discovery set, and brain, liver, adipose and other tissues from various species, all of which failed to show any detectable levels of these hPULCFAs (results not shown). We also could not detect these molecules in various plant tissues or grains, including policosanol extracts which are rich in saturated C28 and longer-chain fatty acids [43,44]. This suggests that the molecules may originate from human-specific metabolic processes, such as specific p450-mediated and/or microbiotic processes. The lack of detection in tumour or normal colonic tissue suggests that the metabolites are not 'tumour-derived markers' and, combined with the high rate of association in stage I cancer, it is not likely that the reduction is the result of tumour burden. Analysis of post-surgery samples is currently in progress to address this question. However, the further reduction of levels observed in some late stage Japanese cases (Figure 6) could be explained if lower levels of the hPULCFAs were indeed indicative of progression rate in this group. It is also important to note that in all control groups reported in this paper, subjects were not colonoscopy-confirmed to be free of tumours or advanced neoplasia. Based upon colonoscopy results by Collins *et al *in average-risk subjects, up to 10% of an asymptomatic population can be positive for advanced neoplasia [45]. Therefore, the ability of these metabolites to discriminate between subjects at risk and not at risk for CRC is likely under-estimated in our results. Studies are currently in progress to evaluate endoscopy-confirmed controls, to assess the effect of treatment on the markers, and to investigate any possible association with various grades of colon pathologies and non-malignant GI disorders as well as other cancers.

Although fatty acids of this length containing hydroxyl groups have never been reported as far as we are aware, they appear to resemble a class of hydroxylated very long-chain fatty acids knows as the resolvins and protectins that originate from the n3 essential fatty acids EPA and DHA, respectively, which are critical in promoting the resolution of acute inflammation. The inability to sufficiently 'resolve' acute inflammation is the leading theory behind the establishment of chronic inflammatory states which underlie multiple conditions including cancer [46] and Alzheimer's Disease [47]. Of particular relevance is the effect of pro-resolution long-chain hydroxy fatty acid mediators on intestinal inflammatory conditions such as irritable bowl disease (IBD), Crohn's Disease, Colitis and colon cancer. Both Resolvin E1 (RvE1) and Lipoxin A4 (LXA4) have been implicated with protective effects against colonic inflammation. RvE1 was shown to protect against the development of 2,4,6-trinitrobenze sulphonic acid-induced colitis in mice, accompanied by a block in leukocyte infiltration, decreased proinflammatory gene expression, induced nitric oxide synthase, with improvements in survival rates and sustained body weight [48]. Similarly, LXA4 analogues have been shown to attenuate chemokine secretion in human colon *ex vivo *[49], and attenuated 50% of genes, particularly those regulated by NFκB induced in response to pathogenically induced gastroenteritis [50]. *In vivo*, LXA4 analogues reduced intestinal inflammation in DSS-induced inflammatory colitis, resulting in significantly reduced weight loss, haematochezia and mortality [50]. Structurally, resolvins and protectins (as well the n6 lipoxins) comprise mono-, di- and tri-hydroxylated products of the parent VLCFAs, catalyzed by various lipoxygenases, cyclooxygenases and p450 enzymes [51-55]. The possibility that the hPULCFAs reported here represent elongation products of these molecules cannot be excluded. Future studies will be required to address the origin, as well as the biological role, if any, that these molecules may play in defending the body against CRC development.

Although we report results from multiple case-control cohorts each having a limited sample size, the average AUC across all the samples reported here was 0.91 ± 0.04, which translates into approximately 75% sensitivity at 90% specificity with little to no disease-stage bias. The real-world screening performance is currently being evaluated through two large ethically approved prospective clinical trials, one in collaboration with the Saskatchewan Cancer Agency and the Saskatchewan Provincial Government (PDI-CT-1; *n *= 5000), and the other with the University of Calgary (PDI-CT-3 *n *= 1500). Clinically relevant questions are being addressed, including correlation between hPULCFAs and CRC in a prospective hospital screening environment, correlation with other non-malignant gastrointestinal disorders (such as IBD, Crohn's and colitis), whether there is any correlation with various stages of neoplasia or polyps and family history and whether subjects with low hPULCFA levels show higher incidence rates of CRC than subjects with 'normal' levels over time.

In summary, we have identified a consistent reduction of novel circulating hPULCFAs in CRC patients which could have considerable implications for CRC diagnosis and screening and possibly prevention and treatment. Adherence to currently recommended screening modalities, namely faecal occult blood testing and colonoscopy, is poor due to a number of factors including public acceptance, risk, cost and available resources. The use of a serum-based test to screen the population for subjects who are high risk would focus endoscopy resources on subjects who need it the most, resulting in a higher detection rate, particularly in early stages of the disease. Given the positive prognosis of early-stage therapeutic intervention, it is tempting to speculate that hPULCFA-based screening could one day result in decreased CRC mortality.

## Conclusions

We have shown that comprehensive non-targeted metabolomics technology based upon high-resolution FTICR mass spectrometry represents a powerful and robust approach for small-molecule biomarker-driven discovery. Accurate mass measurements combined with conventional MS/MS resulted in the rapid identification of key structural characteristics of the novel metabolites discovered and the assignment of putative chemical structures. The subsequent translation of these metabolite biomarker discoveries into an efficient and clinically viable high-throughput semi-quantitative triple-quadrupole platform represents a significant advancement in the clinical implementation of biomarker discoveries. The reduction of systemic hydroxylated ultra-long chain fatty acids in CRC patients raises intriguing biological and aetiological questions given the large numbers of sporadic CRC cases and the heavy influence of lifestyle and diet on risk. Further research is ongoing regarding the potential role(s) these novel molecules play in CRC progression and whether they have any association with previously established risk factors.

## Abbreviations

APC: adenomatous polyposis coli; APCI: atmospheric pressure chemical ionization; AUC: area under the curve; BuOH: butanol; CRC: colorectal cancer; CUR: curtain gas; ESI: electrospray ionization; EtOAc: ethylacetate; FCC: flash column chromatography; FTICR-MS: Fourier transform ion cyclotron resonance mass spectrometry; GS: gas source; HPLC: high performance liquid chromatography; hPULCFA: hydroxylated polyunsaturated ultra long-chain fatty acid; LC: liquid chromatography; LXA4: lipoxin A4; MRM: multiple reaction monitoring; MS/MS: tandem mass spectrometry; mz: mass to charge ratio; NC: nebulizer current; NMR: nuclear magnetic resonance; ppm: part per million; Q-TOF: quadrupole time-of-flight; ROC: receiver-operator characteristic; RvE1: resolving E1; SELDI: surface-enhanced laser desorption ionization; TLC: thin layer chromatography; TQ-MRM: triple-quadrupole MRM; VLCFA: very long-chain fatty acid.

## Competing interests

The following authors are full-time employees and have received salaries from Phenomenome Discoveries Inc, Saskatoon, Canada: Shawn A Ritchie, Pearson W K Ahiahonu, Dushmanthi Jayasinghe, Doug Heath, Jun Liu, Yingshen Lu, Wei Jin, Amir Kavianpour, Yasuyo Yamazaki, Amin M. Khan, Mohammad Hossain, Khine Khine Su-Myat and Paul L Wood. Dayan B Goodenowe is the co-founder, President and CEO, of Phenomenome Discoveries, Inc. Only Dayan B Goodenowe owns shares in the company. Kevin Krenitsky, Ichiro Takemasa, Masakazu Miyake, Mitsugo Sekimoto, Morito Monden, Hisahiro Matsubara and Fumio Numura have no competing financial interests. None of the authors have non-financial competing interests. Phenomenome Discoveries Inc is financing the article processing fees for the manuscript. Shawn A Ritchie and Dayan B Goodenowe are named inventors on submitted patent applications relating to the discoveries disclosed within the manuscript and have both received a salary from Phenomenome Discoveries Inc (the organization named in the patents).

## Authors' contributions

SAR was the group leader, lead author and writer, performed the study design and sample selection, completed all the discovery FTICR-MS data analysis, including statistical analysis, supervised the structural elucidation, MSMS interpretation and overall experimental designs. PWAK performed MS/MS and NMR analysis and interpretation and analysed all comparison MSMS standard data. DJ performed MS/MS and fraction column chromatography enrichment and NMR interpretation. DH developed FTICR-MS analytical methods, including extraction protocols, and helped to develop the TQ-MRM methodology used. JL developed high performance liquid chromatographic methods and analysed samples for HPLC-TOF analysis. YL developed and optimized TQ-MRM methods and analysed validation sample sets. WJ performed and optimized HPLC-TOF and MS/MS analysis and samples, including enriched serum extracts. AK was involved in the optimization of the TQ-MRM methodology, QAQC and analysis of validation (Seracare and Chiba) samples. YY developed the original chromatographic (HPLC) methods used to isolate the hPULCFAs and aided in the experimental design of the Chiba and Osaka sample sets. AMK oversaw the structural elucidation processes including NMR interpretation and MS/MS interpretation. MH aided in MS/MS and NMR data generation and interpretation. KKSM was involved in the biostatistical analysis, including the analysis of HTS data, Fisher analysis and all SAS work. PLW was involved in the interpretation of MS/MS data, NMR data, mechanistic insights into MSMS fragmentation patterns and proposed structural motifs. KK was involved in the sample selection and study design for the genomics collaborative and Seracare Lifesciences sample sets, the selection of matched cases and controls, clinical data organization and verification of disease pathology. IT was involved in the design of clinical trials in Osaka, patient selection and pathology confirmation. MM was involved in the clinical trial design in Osaka, patient selection and discovery analysis, clinical staging, pre-versus post surgery data collection and analysis. MS was the Head of the lower gastroenterological surgery group, Osaka, responsible for patient recruitment and trial design. MM was the group leader at the Osaka Department of Surgery and was involved in the experimental design of the Osaka discovery sample set, data analysis/interpretation and unblinding of data. HM was the head of surgery (for CRC population, Chiba) responsible for the patient enrolment and selection for the Chiba samples. FN was the group leader at Chiba overseeing the entire project, including protocol design and approvals, data analysis and unblinding. DBG was the President and CEO of Phenomenome Discoveries Inc, and oversaw most of the efforts at PDI, was integrally involved in the interpretation of MS/MS data, the development of FTICR methodology, the experimental designs and was also a significant contributor to the format and direction of the manuscript.

## Pre-publication history

The pre-publication history for this paper can be accessed here:

http://www.biomedcentral.com/1741-7015/8/13/prepub

## Supplementary Material

Additional file 1 Fourier transform ion cyclotron resonance mass spectrometry feature data. Click here for file

Additional file 2 **Top 50 discriminating masses (based on student's *t*-test) of each discovery project**. Masses shaded grey were detected in the top 50 in two of the three studies. Indicated are the detected accurate mass, the computationally predicted molecular formula (for masses shaded in grey), the mass difference between the detected mass and mass of the predicted molecular formula in part per million (ppm), the mode of analysis (electrospray ionization; atmospheric pressure chemical ionization), the *P*-value (based on an unpaired student's *t*-test) between the average peak intensity of control subjects versus colorectal cancer (CRC) patients and the average peak intensity ratio between CRC patients and controls. Click here for file

Additional file 3 **Tandem mass spectrometry spectra for biomarker m/z 446**. Click here for file

Additional file 4 **Tandem mass spectrometry spectra for biomarker m/z 448**. Click here for file

Additional file 5 **Tandem mass spectrometry spectra for biomarker m/z 450**. Click here for file

Additional file 6 **Tandem mass spectrometry spectra for biomarker m/z 464**. Click here for file

Additional file 7 **Tandem mass spectrometry spectra for biomarker m/z 466**. Click here for file

Additional file 8 **Tandem mass spectrometry spectra for biomarker m/z 468**. Click here for file

Additional file 9 **Tandem mass spectrometry of hydroxylated polyunsaturated ultra long-chain fatty acids**. Click here for file

Additional file 10 **Purification process to obtain hydroxylated polyunsaturated ultra long-chain fatty acids (hPULCFA) enriched fractions from human serum**. Dried organic extracts of serum were initially purified in a reversed phase flash column chromatography using water/acetonitrile step solvent gradient to obtain semi purified hPULCFA enriched fraction (F9). Several of F9s were combined for a secondary purification step in a normal phase flash column chromatography using hexane/chloroform/methanol step solvent gradient to obtain highly hPULCFA enriched fraction 7 (F7_2). Click here for file

Additional file 11 **Liquid chromatography/mass spectrometry spectra of Stage I fraction 9 (F9) containing a mixture of fatty acids and colorectal cancer biomarkers obtained after fractionating serum extract on reverse phase column**. Click here for file

Additional file 12 Liquid chromatography/mass spectrometry spectra of Stage II fraction 7 (F7) containing approximately 65% enrichment of hPULCFAs. Click here for file
