# Supplementary figures and images for: Reduced levels of hydroxylated, polyunsaturated ultra long-chain fatty acids in the serum of colorectal cancer patients: implications for early screening and detection
Source: BMC Med. 2010 Feb 15;8:13. doi: 10.1186/1741-7015-8-13 (PMC2833138; doi:10.1186/1741-7015-8-13)

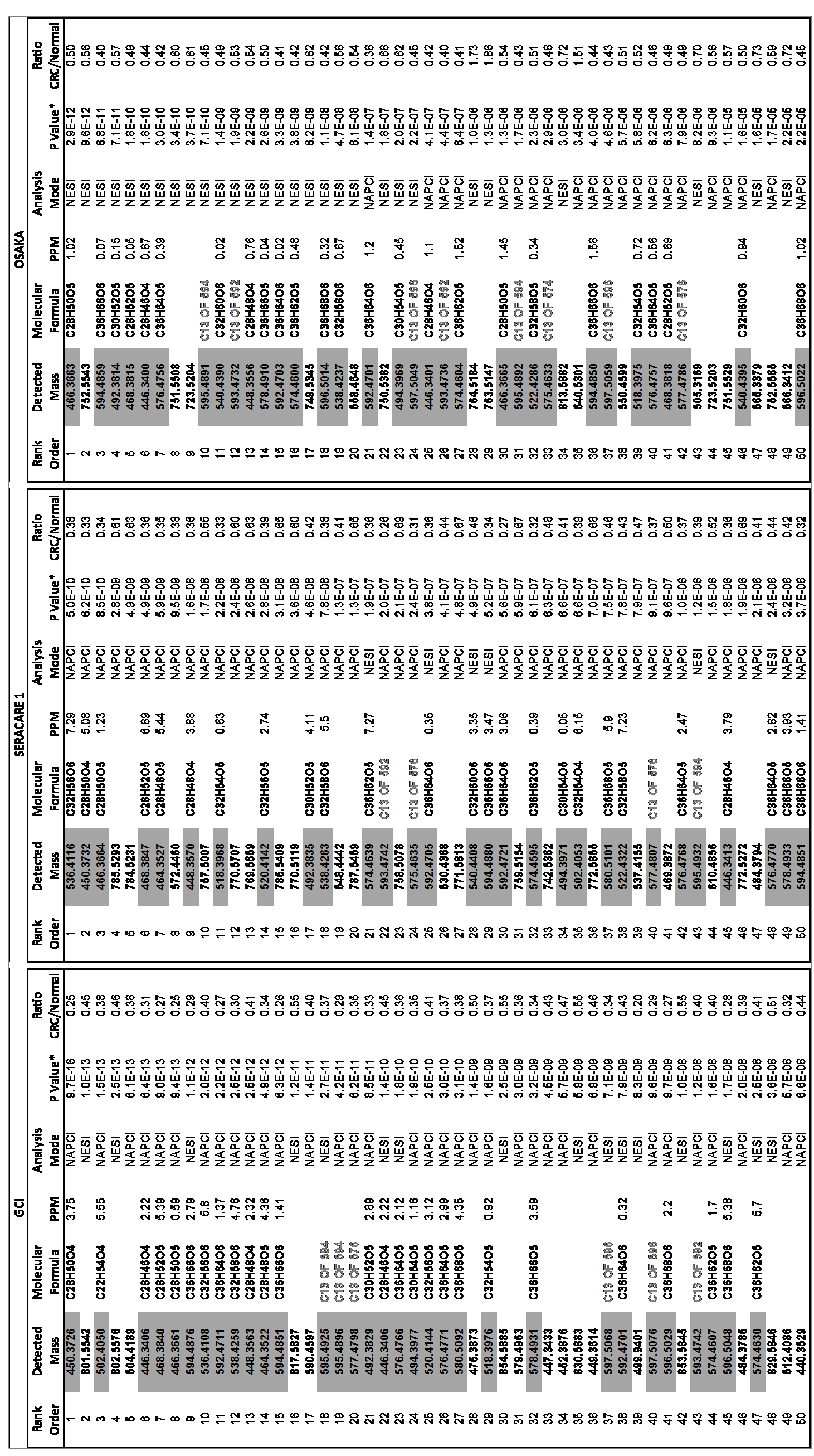

Supplement: Additional file 2 — Top 50 discriminating masses (based on student's t-test) of each discovery project. Masses shaded grey were detected in the top 50 in two of the three studies. Indicated are the detected accurate mass, the computationally predicted molecular formula (for masses shaded in grey), the mass difference between the detected mass and mass of the predicted molecular formula in part per million (ppm), the mode of analysis (electrospray ionization; atmospheric pressure chemical ionization), the P-value (based on an unpaired student's t-test) between the average peak intensity of control subjects versus colorectal cancer (CRC) patients and the average peak intensity ratio between CRC patients and controls. [file 1741-7015-8-13-S2.PNG]

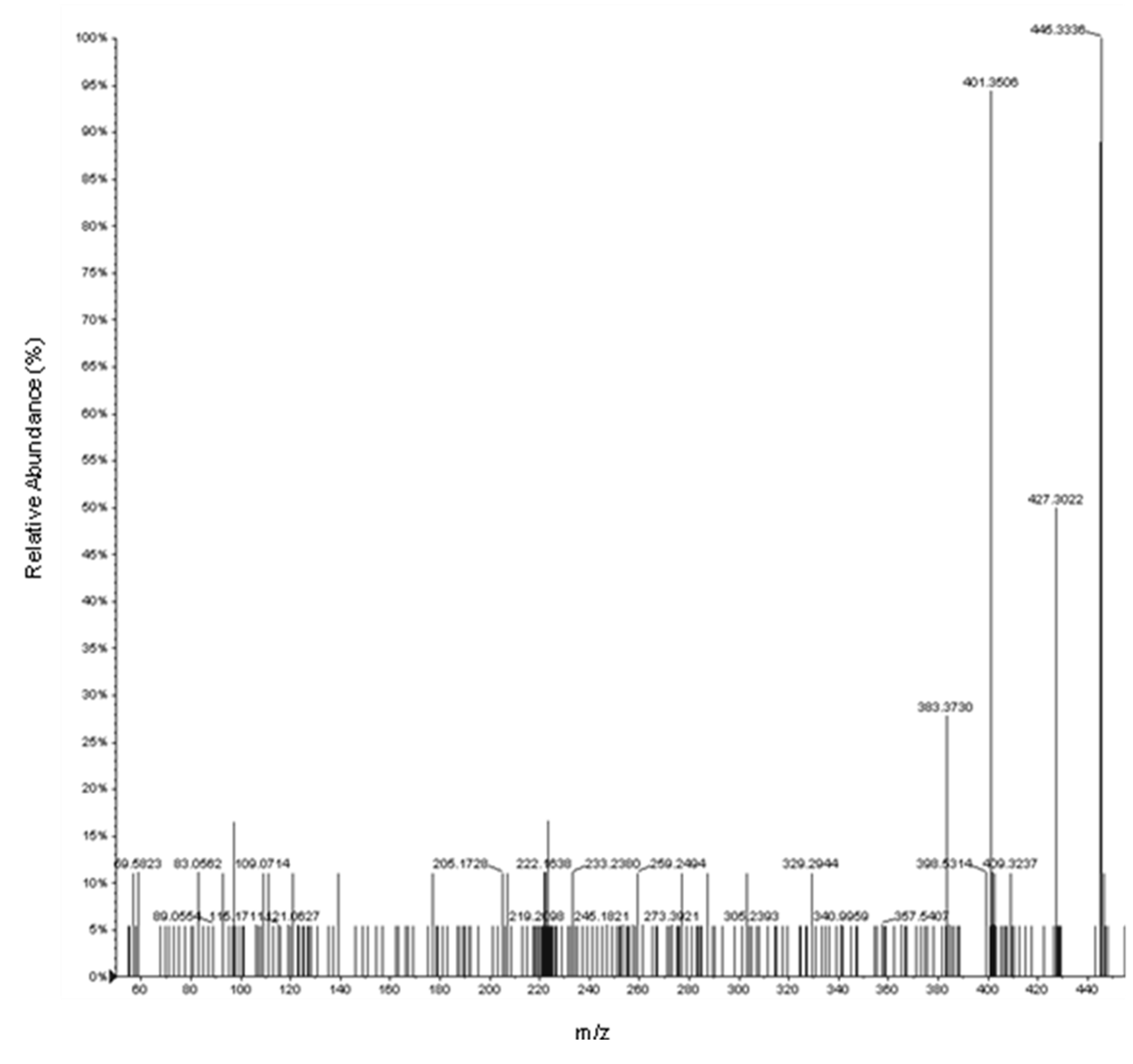

Supplement: Additional file 3 — Tandem mass spectrometry spectra for biomarker m/z 446. [file 1741-7015-8-13-S3.PNG]

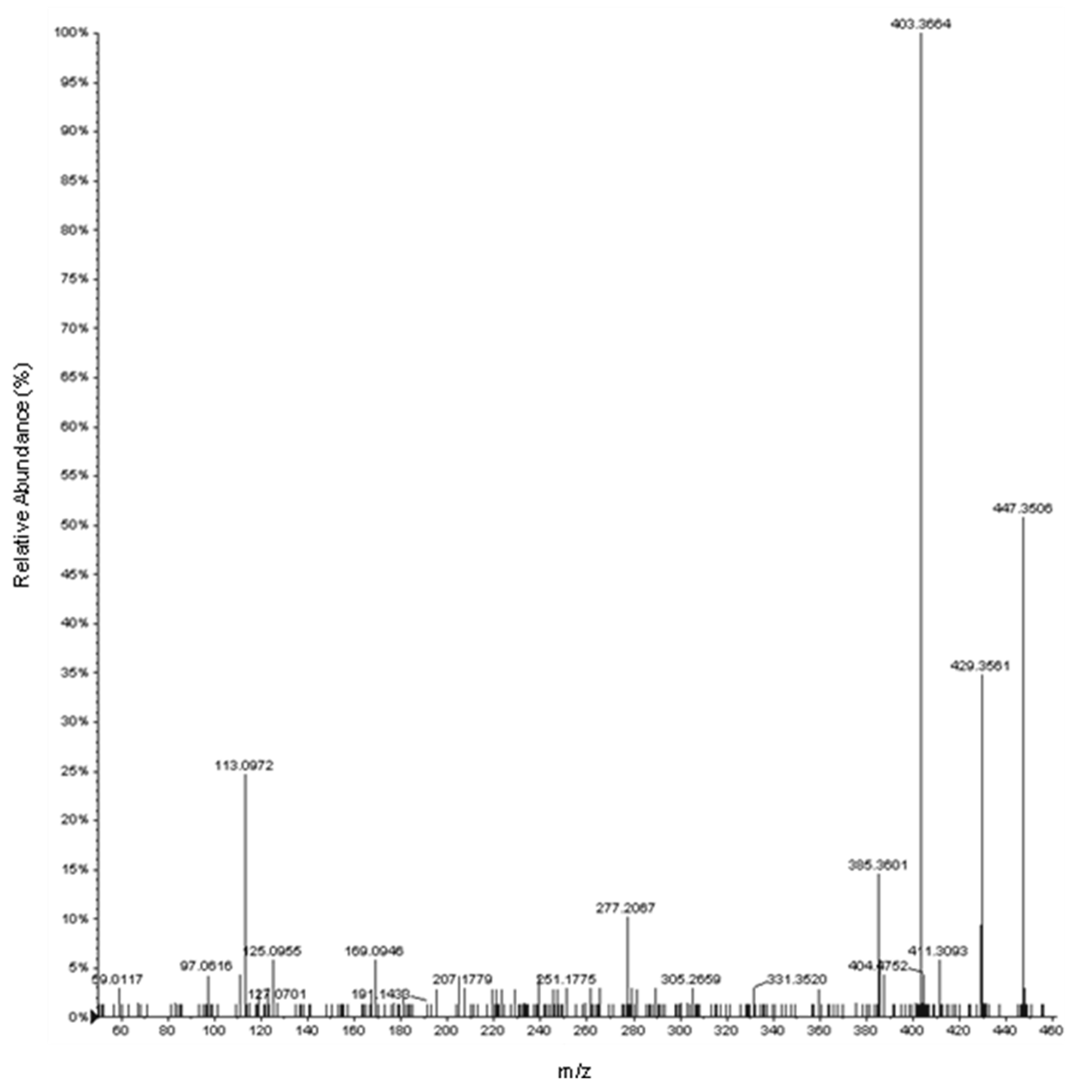

Supplement: Additional file 4 — Tandem mass spectrometry spectra for biomarker m/z 448. [file 1741-7015-8-13-S4.PNG]

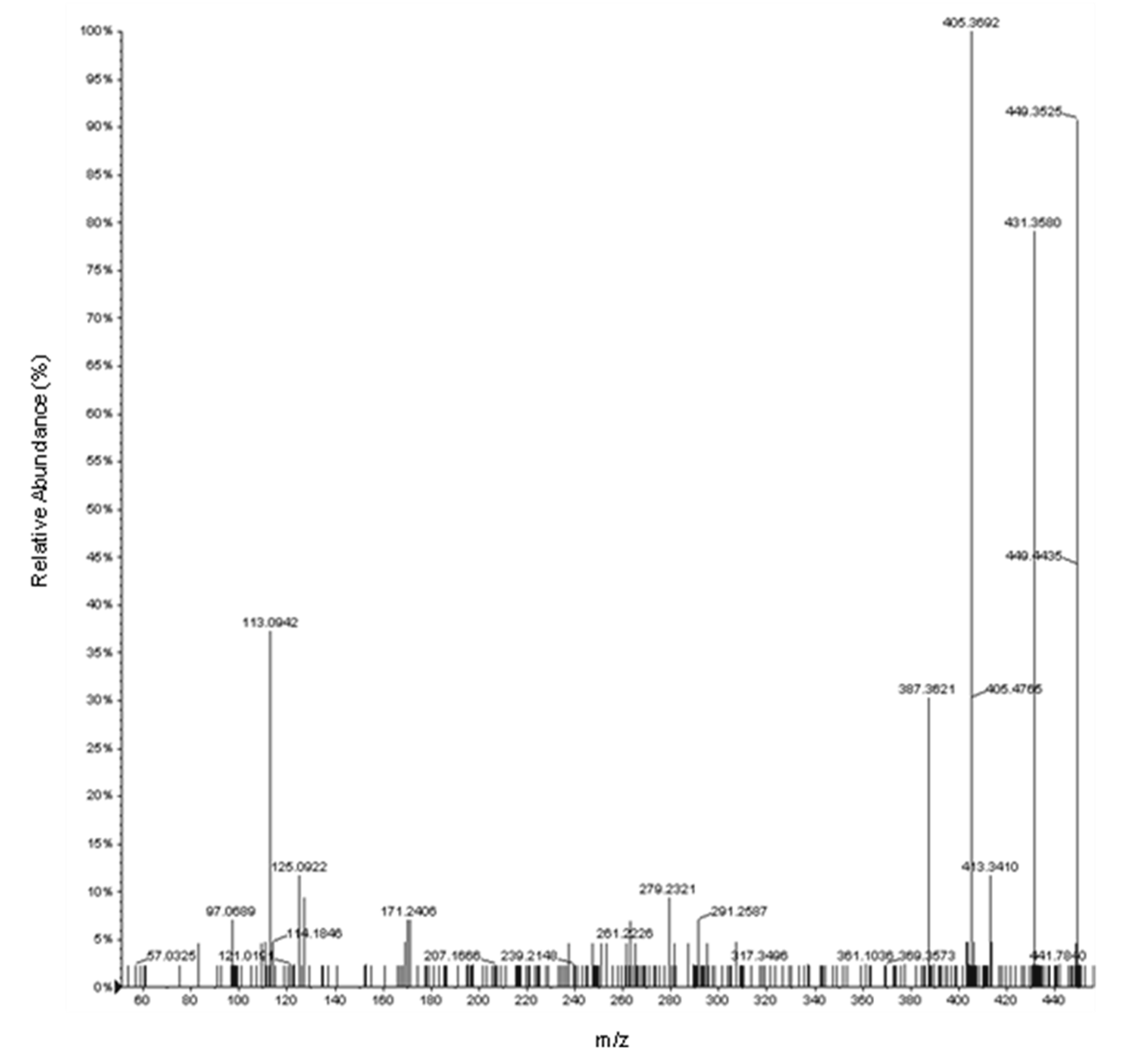

Supplement: Additional file 5 — Tandem mass spectrometry spectra for biomarker m/z 450. [file 1741-7015-8-13-S5.PNG]

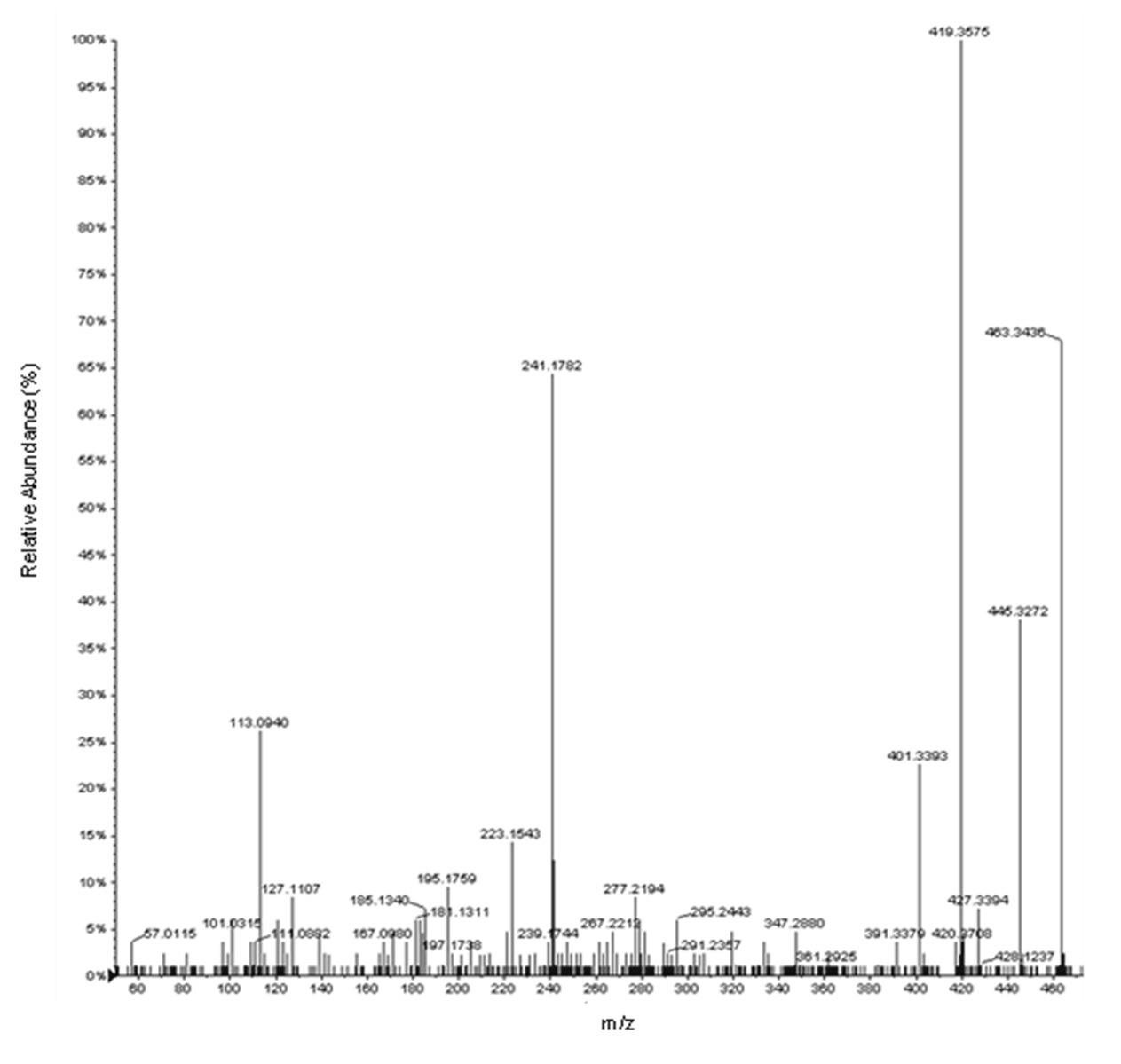

Supplement: Additional file 6 — Tandem mass spectrometry spectra for biomarker m/z 464. [file 1741-7015-8-13-S6.PNG]

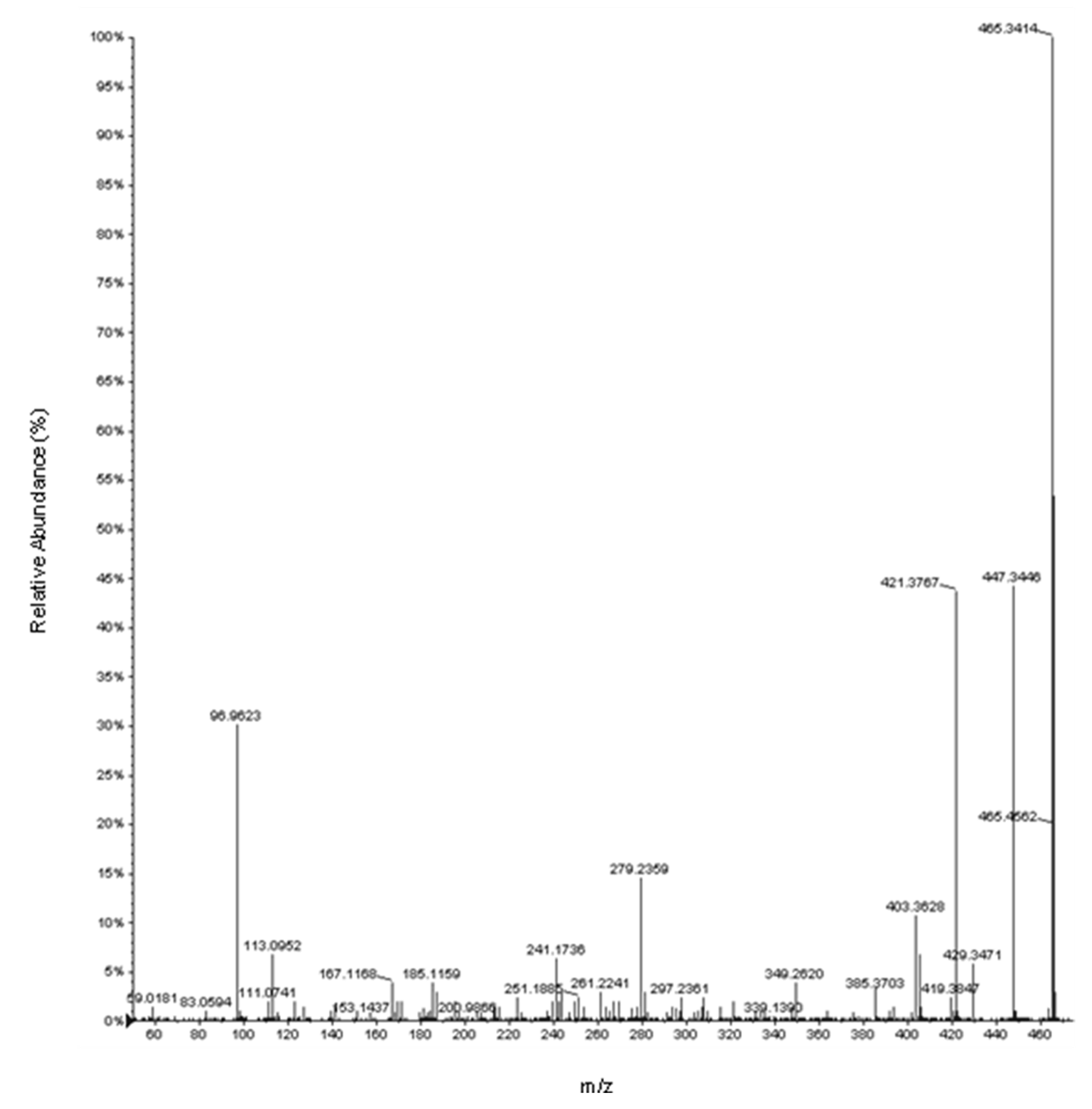

Supplement: Additional file 7 — Tandem mass spectrometry spectra for biomarker m/z 466. [file 1741-7015-8-13-S7.PNG]

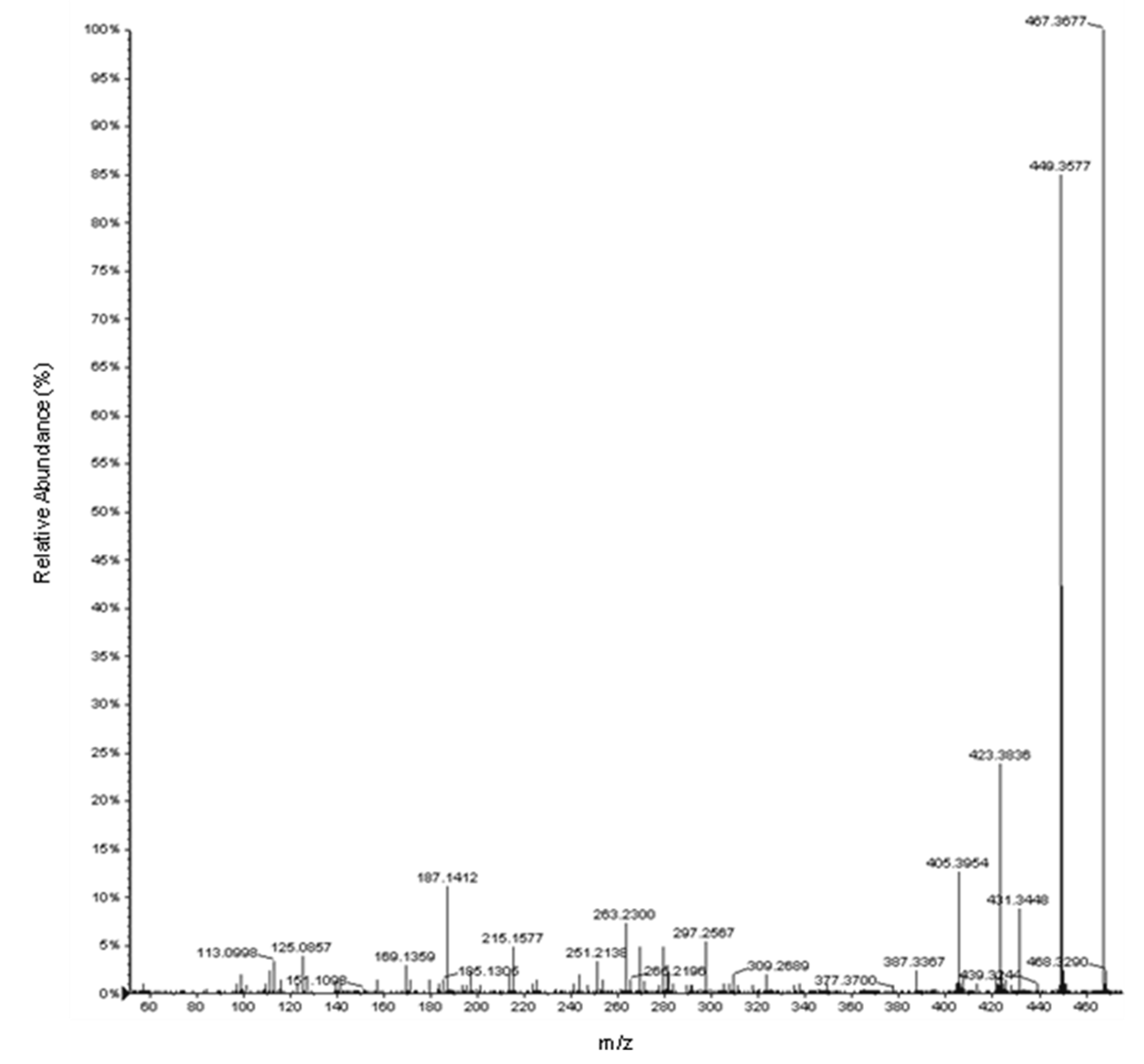

Supplement: Additional file 8 — Tandem mass spectrometry spectra for biomarker m/z 468. [file 1741-7015-8-13-S8.PNG]

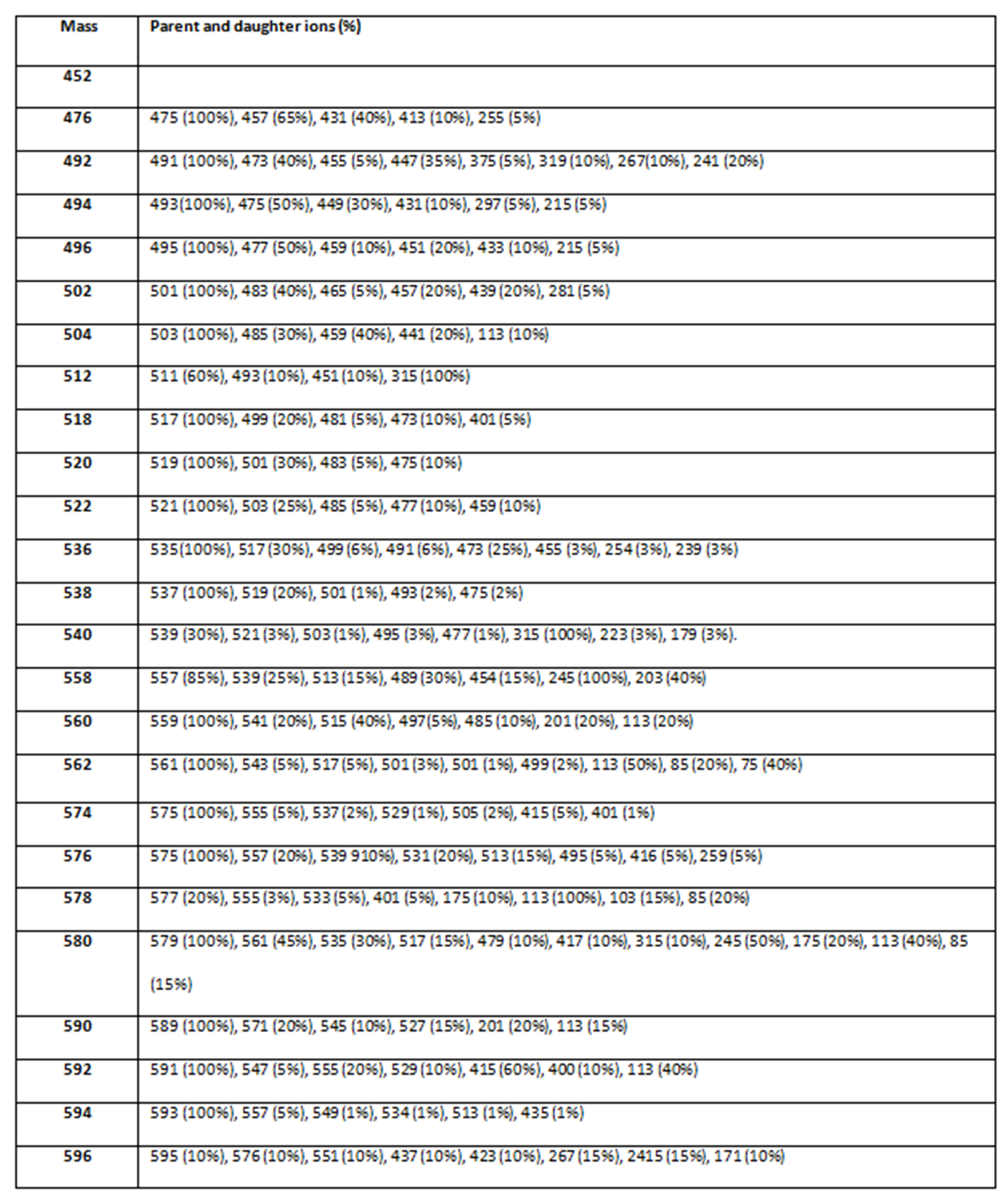

Supplement: Additional file 9 — Tandem mass spectrometry of hydroxylated polyunsaturated ultra long-chain fatty acids. [file 1741-7015-8-13-S9.PNG]

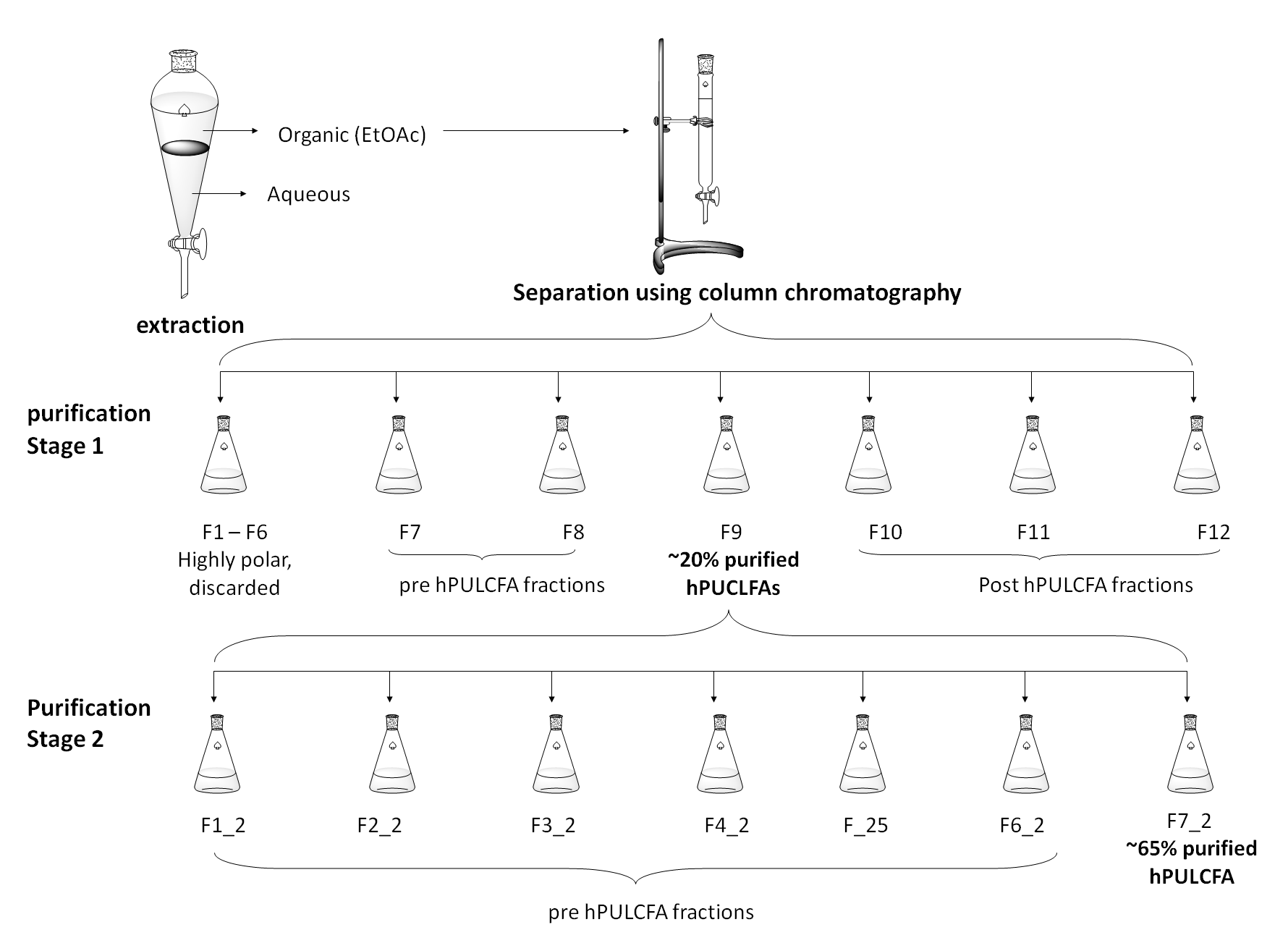

Supplement: Additional file 10 — Purification process to obtain hydroxylated polyunsaturated ultra long-chain fatty acids (hPULCFA) enriched fractions from human serum. Dried organic extracts of serum were initially purified in a reversed phase flash column chromatography using water/acetonitrile step solvent gradient to obtain semi purified hPULCFA enriched fraction (F9). Several of F9s were combined for a secondary purification step in a normal phase flash column chromatography using hexane/chloroform/methanol step solvent gradient to obtain highly hPULCFA enriched fraction 7 (F7_2). [file 1741-7015-8-13-S10.PNG]

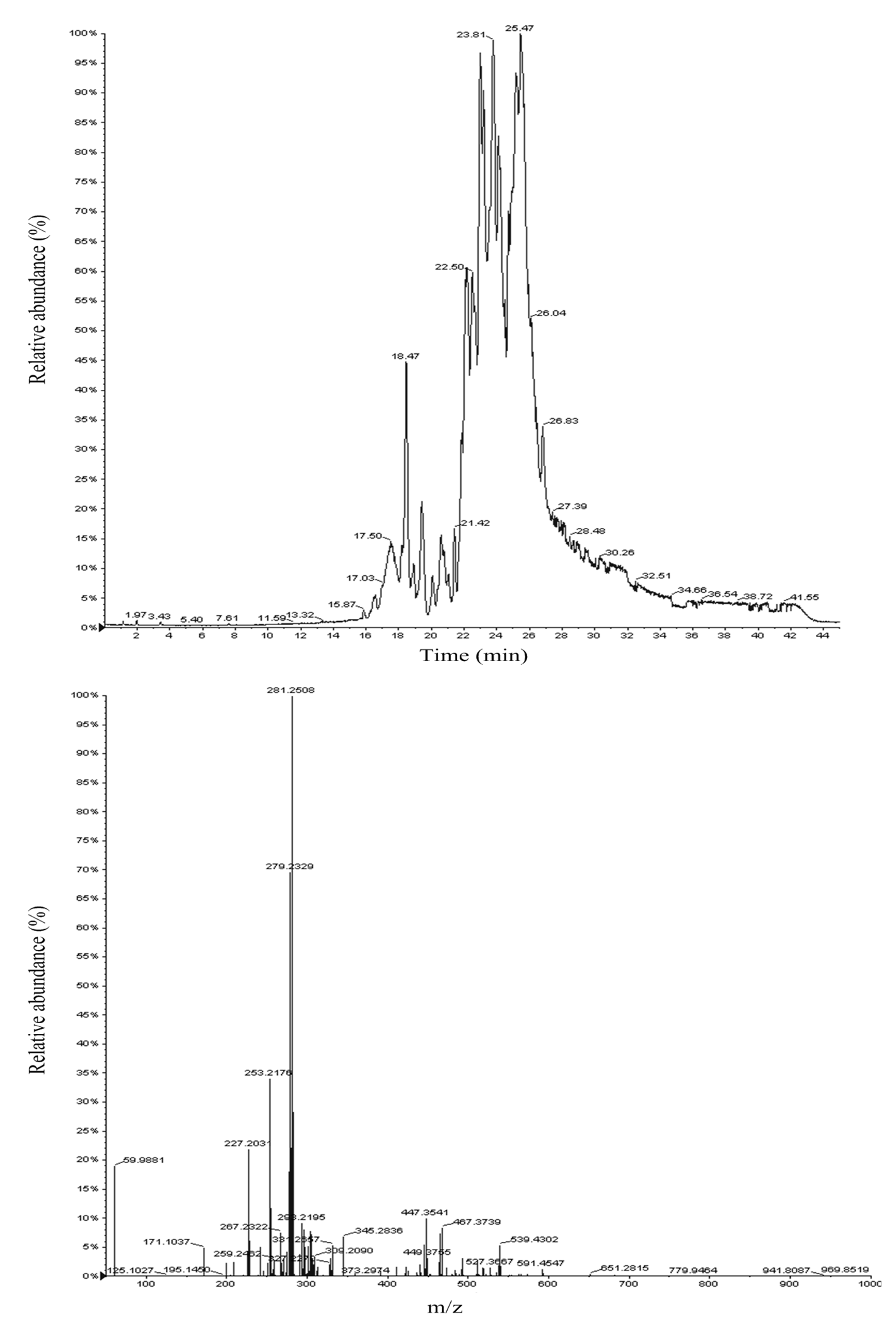

Supplement: Additional file 11 — Liquid chromatography/mass spectrometry spectra of Stage I fraction 9 (F9) containing a mixture of fatty acids and colorectal cancer biomarkers obtained after fractionating serum extract on reverse phase column. [file 1741-7015-8-13-S11.PNG]

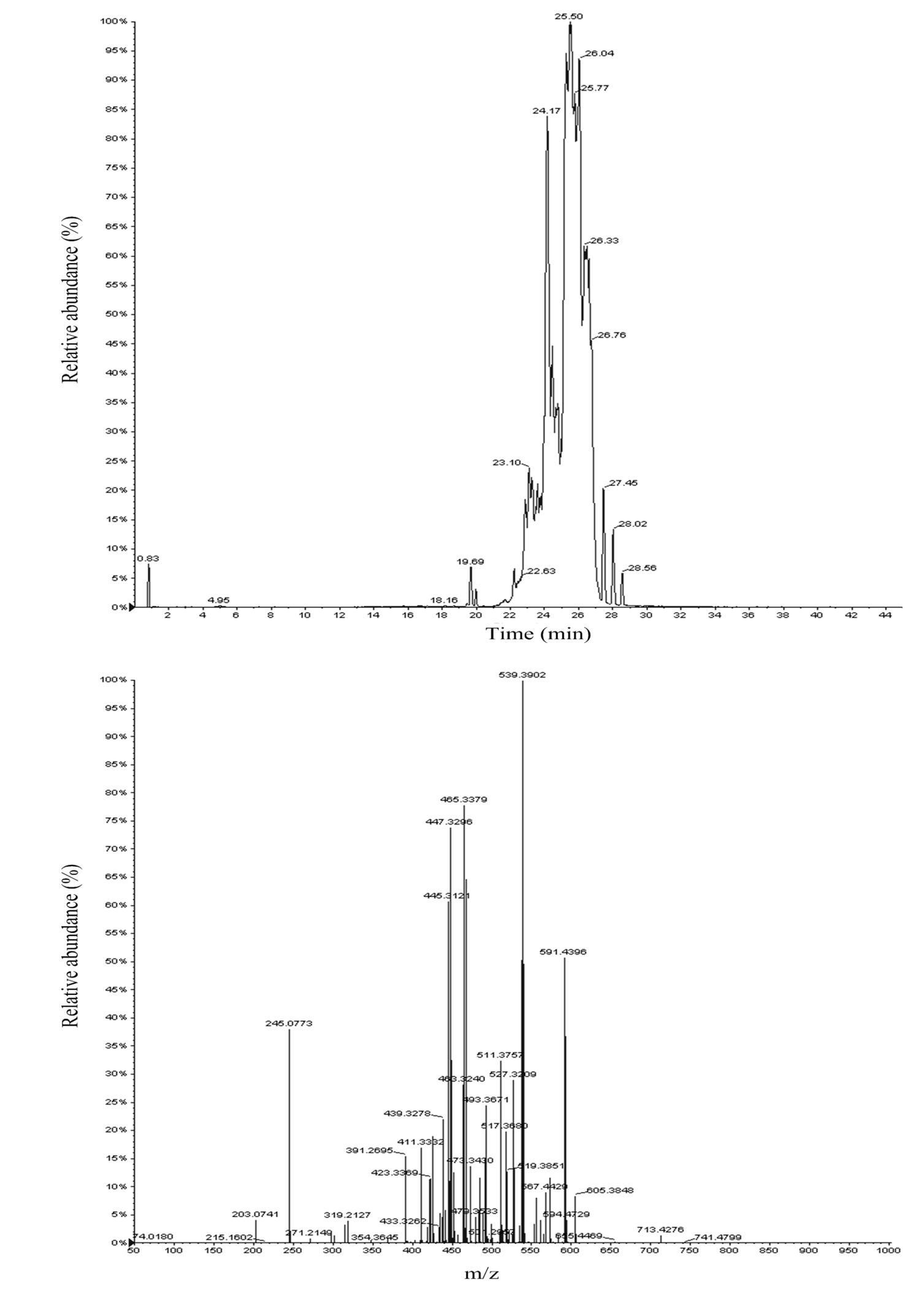

Supplement: Additional file 12 — Liquid chromatography/mass spectrometry spectra of Stage II fraction 7 (F7) containing approximately 65% enrichment of hPULCFAs. [file 1741-7015-8-13-S12.PNG]
